# Supplementary material for: Evaluation of hypofractionated adaptive radiotherapy using the MR Linac in localised pancreatic cancer: protocol summary of the Emerald-Pancreas phase 1/expansion study located at Oxford University Hospital, UK
Source: BMJ Open. 2023 Sep 14;13(9):e068906. doi: 10.1136/bmjopen-2022-068906 (PMC10503372; doi:10.1136/bmjopen-2022-068906)
Supplement: Supplementary data [file bmjopen-2022-068906supp001.pdf]

Eligibility criteria for entry into the main study

Inclusion criteria:

A patient will be eligible for inclusion in this study if all of the following criteria apply.

1. Participants must be fit and scheduled to receive MR guided radiotherapy for pancreatic cancer. There are no specific restrictions on tumour size, number or interval from diagnosis.
2. Localised pancreatic cancer, which may be
    a. locally advanced and inoperable pancreatic cancer
    b. inoperable on medical grounds
    c. operable, but patient declines surgery
    d. locally recurrent pancreatic cancer
3. Histologically proven pancreatic ductal adenocarcinoma or cytological proven pancreatic malignancy. Where histology/cytology is 'suspicious' MDT should confirm that it is appropriate to treat as malignancy.
4. Male or Female, aged 16 years or above.
5. Life expectancy of at least 6 months.
6. ECOG performance status 0- 1.
7. Haematological and biochemical indices within defined ranges:

Lab Test	Value required
Haemoglobin (Hb)	≥ 8.0 g/dL
Platelet count	≥50 x 10^9/l;
Neutrophils	≥ 1.0 x 10^9/l.
Total bilirubin	≤ 1.5 x IULN
AST(SGOT) or ALT(SGPT)	≤ 3.0 x IULN

8. Able (in the investigators' opinion) and willing to comply with all study requirements for the duration of the study.
9. Willing and able to give informed consent.

Exclusion criteria:

A patient will not be eligible for the trial if any of the following apply:

1. Patients with specific MRI exclusion criteria – metallic implants, shrapnel, claustrophobia or other expected intolerance of prolonged (up to 90 minutes) stay in MRI scanner.
2. Prior radiotherapy to the upper abdomen
3. Pregnant or breast-feeding women, or women of childbearing potential unless effective methods of contraception are used. Male patients who do not agree to use a condom during RT treatment and for three months after or who are not surgically sterile.
4. Distant metastatic disease or local disease that cannot be encompassed in the SABR field.
