# Supplementary material for: Evaluation of hypofractionated adaptive radiotherapy using the MR Linac in localised pancreatic cancer: protocol summary of the Emerald-Pancreas phase 1/expansion study located at Oxford University Hospital, UK
Source: BMJ Open. 2023 Sep 14;13(9):e068906. doi: 10.1136/bmjopen-2022-068906 (PMC10503372; doi:10.1136/bmjopen-2022-068906)
Supplement: Supplementary data [file bmjopen-2022-068906supp002.pdf]

SCHEDULE OF ASSESSMENTS

**Procedure** *(grey shading denotes SoC activities)*	**Pre-screening** Within 3 months of RT #1	**Screening & baseline** Within 28 Days of planning scan	**Planning scan visit**	**RT fraction[14]**					**Post radiotherapy**							**Progress-ion** (if applicable)	**Early Withdra-wal** (if applicable)	**End of study[19] (+/-1 months)**
				**#1**	**#2**	**#3**	**#4**	**#5**	**+1 weeks[16] (+/- 4 days)**	**3 weeks[17] (+/- 1 wk)**	**6 weeks[17] (+/- 2 wks)**	**3 months[17] (+2 wks)**	**6 months[17] (+/- 1 mon)**	**12 months[17] (+/- 1 mon)**	**18 months[17] (+/- 1 mon)**			
**CT Scan TAP [1]**	X											X	X	X		X		
**Informed consent [2]**		X																
**Demographics[3]**		X																
**Baseline sign & symptoms[4]**		X																
**Medical history**		X																
**Haematology[5]**		X								X		X						
**Biochemistry[6,7]**		X								X		X						
**Ca19.9 tumour marker**		X								X		X						
**Urine pregnancy test (WOCBP only)**		X																
**Clinical review & disease assessment [8]**		X							X	X	X	X	X	X	X		X	
**Performance status**		X		X[15]					X		X	X	X	X	X			
**AE review [9]**			X[21]	X	X	X	X	X	X	X	X	X	X	X	X		X	X
**DLT review**				X	X	X	X	X	X	X	X	X					X[18]	
**Late-onset severe toxicities review**													X	X	X		X[20]	X
**Planning scan (0.35T MRI)**			X															
**MR Linac treatment [11]**				X	X	X	X	X										
**Optional research blood sample[12]**		X								X		X						
**Data capture: PFS, survival status, resection status, restart of chemotherapy & locoregional failure[13]**												X	X	X [19]	X	X	X [19]	X [19]
**Reason for withdrawal**																	X	

1. Standard of care (SOC) diagnostic CT and optional MRI are used as baseline imaging and whole-body PET-CT if performed in routine care. Further CT scans (& MRI, PET-CT where applicable) at 3, 6 & 12 months follow-up and if relapse/progression occurs are also standard of care. Scans and clinical report to be pseudonymised with trial subject ID and transferred to OU. Scan images and report need to be transferred to the OU if scan carried out at another trust.
2. See section 4.5 for further information on informed consent process.
3. Demographic details to include age and sex
4. Baseline Sign and Symptoms: provide date of onset, event diagnosis (if known) or sign/symptom, severity, time course. Terms should be specific medical terms according to NCI CTCAE version 5. Please avoid using abbreviations, combined terms e.g. nausea and vomiting and ambiguous terms e.g. deranged, abnormal.
5. Haematology: Full Blood Count.
6. eGFR at baseline only.
7. Biochemistry: sodium, potassium, urea and electrolytes, creatinine, ALT or AST, Bilirubin, Albumin, alkaline phosphatase.
8. As considered appropriate by clinician. In follow-up this should include disease assessment for progression
9. AE assessment may be undertaken face to face, by telephone or audio/video call through the internet.
10. The planning scan is required as a routine part of clinical care, for the study an additional set of research images may be acquired during the planning scan.
11. MR Linac treatment will be given in 5, 3 or 1 fractions as per dose selection process detailed in section 10.0 & 10.1.
12. Optional Research Blood Sample: For patients who have consented: Research bloods to be taken pre-treatment, 3 week and 3 months post RT.
13. For participants who have not reached 24 months follow-up at study closure this data will be collected at an earlier timepoint, at least 3 months after start of RT and as close as possible to study closure.
14. Fractions may be missed/delayed at Investigators' discretion
15. ECOG status prior to fraction 1.
16. From the end of radiotherapy
17. From start of radiotherapy
18. If early withdrawal is within 3-month DLT collection window
19. Evaluations to be completed at end of study, 24 months (from RT Fraction #1) follow-up or death
20. If early withdrawal is after 3-month DLT Collection window.
21. The AE Review at the Planning Scan Visit can be performed with the RT Fraction #1 AE review, as long as the review includes AE assessment from the Planning Scan Visit date.
