# Supplementary material for: Evaluation of hypofractionated adaptive radiotherapy using the MR Linac in localised pancreatic cancer: protocol summary of the Emerald-Pancreas phase 1/expansion study located at Oxford University Hospital, UK
Source: BMJ Open. 2023 Sep 14;13(9):e068906. doi: 10.1136/bmjopen-2022-068906 (PMC10503372; doi:10.1136/bmjopen-2022-068906)
Supplement: Author's manuscript [file bmjopen-2022-068906.draft_revisions.pdf]

BMJ Open is committed to open peer review. As part of this commitment we make the peer review history of every article we publish publicly available.

When an article is published we post the peer reviewers' comments and the authors' responses online. We also post the versions of the paper that were used during peer review. These are the versions that the peer review comments apply to.

The versions of the paper that follow are the versions that were submitted during the peer review process. They are not the versions of record or the final published versions. They should not be cited or distributed as the published version of this manuscript.

BMJ Open is an open access journal and the full, final, typeset and author-corrected version of record of the manuscript is available on our site with no access controls, subscription charges or pay-per-view fees (http://bmjopen.bmj.com).

If you have any questions on BMJ Open's open peer review process please email info.bmjopen@bmj.com

BMJ Open

Evaluation of hypofractionated adaptive radiotherapy using the MR Linac in localised pancreatic cancer (Emerald-Pancreas): Phase 1/expansion study

Journal:	*BMJ Open*
Manuscript ID	bmjopen-2022-068906
Article Type:	Protocol
Date Submitted by the Author:	06-Oct-2022
Complete List of Authors:	Teoh, Suliana; University of Oxford Department of Oncology; Oxford University Hospitals NHS Foundation Trust<br>Ooms, Alexander; University of Oxford, Nuffield Department of Orthopaedics, Rheumatology and Musculoskeletal Disorders<br>George, Ben; GenesisCare UK<br>Owens, Rob; Oxford University Hospitals NHS Foundation Trust; GenesisCare UK<br>Chu, Kwun-Ye; University of Oxford Department of Oncology; Oxford University Hospitals NHS Foundation Trust<br>Drabble, Joe; GenesisCare UK<br>Robinson, Maxwell; Oxford University Hospitals NHS Foundation Trust<br>Parkes, Matthew; Oxford University, Oxford Clinical Trials Research Unit (OCTRU)<br>Swan, Lynda; University of Oxford Department of Oncology<br>Griffiths, Lucinda; University of Oxford Department of Oncology<br>Sivakumar, Shivan; University of Oxford Department of Oncology<br>Good, James; GenesisCare UK<br>Maughan, Tim; University of Oxford Department of Oncology; Oxford University Hospitals NHS Foundation Trust<br>Mukherjee, Somnath; University of Oxford Department of Oncology; Oxford University Hospitals NHS Foundation Trust
Keywords:	Magnetic resonance imaging < RADIOTHERAPY, Hepatobiliary tumours < ONCOLOGY, Radiation oncology < RADIOTHERAPY

SCHOLARONE™
Manuscripts

Title: Evaluation of hypofractionated adaptive radiotherapy using the MR Linac in localised pancreatic cancer (Emerald-Pancreas): Phase 1/expansion study

Authors: S Teoh[1,2], A Ooms[3], B George[4], R Owens[2,3], KY Chu[1,2], J Drabble[4], M Robinson[2], M Parkes[3], L Swan[1], L Griffiths[1], S Sivakumar[1], J Good[4], T Maughan[1], S Mukherjee[1,2]

[1] Department of Oncology, University of Oxford, OX3 7DQ, UK
[2] Oxford University Hospitals NHS Foundation Trust, OX3 7LE, UK
[3] Nuffield Department of Orthopaedics, Rheumatology and Musculoskeletal Disorders, University of Oxford, OX3 7LD, UK
[4] GenesisCare UK, OX4 6LB, UK

Corresponding author: Dr Suliana Teoh

Corresponding address:
Department of Oncology
University of Oxford
Old Road Campus Research Building
Roosevelt Drive
Oxford OX3 7DQ

Mobile: +44 7921855863

Email: suliana.teoh@oncology.ox.ac.uk

Keywords: Pancreatic cancer, online adaptive radiotherapy, MR-guided radiotherapy, SABR

Word count: 3880

For peer review only

Abstract

Introduction:
Online adaptive magnetic resonance guided radiotherapy (MRgRT) allows for dose escalation to pancreatic cancer while sparing surrounding critical organs. We seek to evaluate the safety of delivering hypofractionated five-, three- and single fraction MR-guided stereotactic ablative radiotherapy (SABR) to the pancreas.

Methods and analysis:
This is a single centre three-arm phase 1 non-randomised safety study. Patients with localised pancreatic cancer will receive either 50Gy in five- (BED_{10} =100Gy), 39Gy in three- (BED_{10} =90Gy), or 25Gy in a single fraction (BED_{10} =87.5Gy) MR-guided daily online adaptive radiotherapy. Each fractionation regime will be assessed as independent cohorts to determine tolerability, assessed continuously using Bayesian conjugate posterior beta distributions. The primary endpoint of the study is to establish the safety of five-, three- and single fraction MR-guided hypofractionation SABR in localised pancreatic cancer by assessing dose-limiting toxicities. Secondary endpoints include overall survival, progression free survival, local control rates, overall control rate, resection rates, long-term toxicities and freedom from second line chemotherapy. This study plans to also explore imaging and immune biomarkers that may be useful to predict outcome and personalise treatment. The trial will recruit up to 60 patients with a safety run-in.

Ethics and dissemination: The trial is approved by the West Midlands - Black Country Research Ethics Committee 22/WM/0122.The results will be disseminated via conference presentations, peer-reviewed scientific journals and submission to regulatory authorities.

Trial registration number: ISRCTN10557832

Keywords:
Pancreatic cancer, online adaptive radiotherapy, MR-guided radiotherapy, SABR

Strength and Limitations

- This is a phase I safety study evaluating online adaptive extreme hypofractionated radiotherapy using novel MR-guided-radiotherapy (strength).
- There is continuous monitoring of the primary outcome of dose limiting toxicity using conjugate posterior beta distribution (strength).
- Longer term outcome data is limited due to follow-up period of up to 24 months (limitation).
- The trial has a non-comparative design (limitation).
- All three fractionation regimes will be considered as three separate cohorts and therefore outcome could be that three different SABR prescriptions are safe to deliver in pancreatic cancer (strength).

For peer review only

Background

The role of radiotherapy in patients with inoperable pancreatic cancer is controversial due to its high propensity to metastasize. Meaningful benefit of local control is challenging to quantify when most patients die of distant metastatic disease. Nevertheless, not all patients with adenocarcinoma of the pancreas develop distant metastatic disease. A third of these patients die mainly of local progression [1]. As systemic treatment improves, local control is likely to be increasingly important. Stereotactic ablative radiotherapy (SABR) where an ablative dose of RT is delivered to a small volume in one to five fractions, has been shown to achieve local control rates as high as 80-100%[2-8], compared to about 50-70% reported in CRT trials [9 10]. Initial SABR experiences for treatment of locally advanced pancreatic cancer with ablative doses of radiation had reported high rates of toxicity, particularly with regimens delivered in less than 5 fractions [5 6 11].

There are a number of challenges in delivering highly conformal radiotherapy in pancreatic cancer. Firstly, tumour and organs-at-risk (OAR) visualisation during treatment setup is limited with CT-based imaging. Moreover, the proximity of pancreatic tumours to highly mobile radiosensitive organs such as the duodenum, stomach and bowel means delivering high doses while sparing these organs is difficult. A number of studies have explored the use of SABR to improve outcome. Of concern is the toxicity rate observed in some of these studies. Hoyer et al treated patients with 45Gy in 3 fractions and reported 5 out of 22 patients developing severe mucositis or perforation[11]. Not all patients had on-board CT-imaging for radiotherapy setup. Similarly, Liauw et al reported up to 27% grade 3 and above late toxicities following three-fraction SABR [6]. In both cases large PTV margin and no motion management was utilised. Schellenberg et al evaluated concurrent gemcitabine with single fraction 25 Gy SABR for LAPC and reported freedom from local progression was 88% at 1 year. However, they reported 15% grade 2 ulcers and one patient with a duodenal perforation [12].

Daily online adaptive magnetic resonance imaging (MRI)-guided radiotherapy (MRgRT) is a novel method of delivering radiotherapy that could potentially allow for dose escalation without exceeding dose to the organs-at-risk (OAR). MRI provides superior visualisation of organs such as the stomach, duodenum, and bowel as well as improved tumour identification. Online adaptive MRgRT gives the opportunity to adapt treatment plans daily to account for inter-fraction variability of these organs to maintain target coverage while sparing high doses to OAR [13], Teoh, et al ASTRO 22 poster). Furthermore, the ability to track the tumour during treatment delivery means 'real-time' intra-fraction monitoring is possible.

Early data from retrospective studies shows that dose escalation to 50Gy in 5 fractions using online adaptive MRgRT is safe and may result in improved survival (ref). A retrospective analysis of 42 locally advanced pancreatic cancer patients treated by MR-guided adaptive RT at four institutions demonstrated that high-dose SABR or hypofractionated radiation therapy delivered using daily adaptive dose planning has the potential to further improve overall survival [14] . A control group of 19 patients treated to more conventional radiation doses without frequent dose adaptation showed a median survival of 14.8 months, while patients treated to high radiation doses (n=23, maximum BED10 of >90 Gy) under daily or almost daily adaptive re-planning had an estimated median survival of 27.8 months (p=0.005). Increased radiation dose delivery using daily dose adaptation was correlated with less grade 3 toxicity (0% in the high dose group vs 15.8% in patients treated to lower radiation doses without dose adaptation). Hall et al, summarised the current published experience for MRgRT in pancreatic cancer and found local control rates of between 77-88% at 1 year with toxicity rates of less than 10% (n=141 from 6 studies mostly retrospective) [15]. Chuong et al, retrospectively analysed 50 pancreatic cancer patients treated with 50Gy in 5 fraction using online daily adaptation on the ViewRay MRlinac and found one-year, and 2-year estimated local control were 97.8%, and 88.9%, respectively. Median survival was 21 months (1 and 2-year OS 87.9% and 50%) (ESTRO 2021) [16].

Pancreatic adenocarcinoma features both local and systemic immunosuppression[17]. This enables immune escape and establishment of the tumour both at the primary and distant sites. Whether or not ablative radiation doses to the primary tumour can induce systemic tumour immunity in pancreatic cancer is unknown. In murine pancreatic ductal adenocarcinoma (PDAC) models, SABR has been shown to induce immunogenic cell death (ICD) and promote tumour cell antigen presentation resulting

For peer review only

in activation of tumour-specific T cells [18]. Mills et al evaluated the immune response from histopathological samples of patients treated with SABR (25Gy in 5 fractions) pre-operatively vs surgery alone and found that SABR reduced PDAC cell density, induced ICD and increased the proportion of PD-1+ T cells. However, despite these finding, intra-tumour T cell levels remain low and outnumbered by myeloid suppressors[19]. While SABR to an unselected population of LAPC patients is unlikely to significantly impact survival outcomes, there is potential for a multi-modality approach with immunotherapy, particularly when combined with biomarkers suggestive of a locally aggressive rather than metastatic phenotype.

The Emerald-Pancreas trial will evaluate the safety of delivering five-, three and single fractions SABR to the pancreas using online adaptive MRgRT. Shorter fractionation schedules are more convenient for patients and increase access to and cost-effectiveness of the scarce and expensive resource of an MRgRT machines. Being able to achieve high rates of local control of the primary tumour with minimal side effects and limited impact on patient lives would be a great advantage for patients. It will also enable greater access for systemic therapies, including conventional chemotherapy and novel immunotherapies, to develop more effective long term control regimens to combat this highly aggressive disease.

Methods/Design

The aim of the trial is to assess safety of extreme hypofractionation of SABR using MRgRT in pancreatic cancer. The five-, three- and single fraction MRgRT treatments will be assessed as independent cohorts to determine if each one is tolerable. Tolerability will be assessed using conjugate posterior beta distributions.

Trial design

This is a three-arm, non-randomised, open, safety study. There are three phases of recruitment to the study: an initial safety run-in, a focussed recruitment phase, and an expansion phase (see Figure 1).

In the initial safety run-in, three patients will be recruited into the five-fraction regimen. The three-fraction regimen will open immediately once three patients have been recruited into five-fraction regimen. Once three patients have been accrued to the three-fraction regimen, the single fraction regimen can open immediately. Recruitment to the one- and three-fractions regimens will pause while waiting for 3 months dose limiting toxicity (DLT) follow-up from the first three patients in each cohort. It is possible both the single and three fraction regimens will be paused at the same time. Any patients recruited whilst both one- and three-fractions regimens are paused will be assigned the five-fraction regimen.

Following the safety run-in, the focussed recruitment will start. Recruitment will continue in cohorts of 3 alternating between the three- and one-fraction regimens until a total of 12 patients in the one- and three-fraction regimens have been recruited. Recruitment to this phase will be continuous. After 3 patients' full DLT follow-up data in any regimen, the beta binomial model for that regimen can be run to assess the safety stopping rule in the event of a DLT.

For the remainder of the trial, the expansion phase recruitment will be in cohorts of 1 to all three regimens and recruitment will be continuous, alternating between one-, three-, and five-fractions. Where plans that meet dose constraints are unable to be generated for single fraction regimens that patient will be assigned to the three-fraction regimen or alternatively the five-fraction regimen if recruitment to the three-fraction cohort is paused or closed. Where plans that meet dose constraints are unable to be generated for three-fraction regimens, that patient will be assigned to the five-fraction regimen. These patients will be included in the main analysis for the regimen they are treated in. A sensitivity analysis will be performed excluding these patients as they may be at higher risk of toxicity. Figure 2 shows the trial schema and overview of follow-up. The duration of the study will be up to 24 months from patient starting radiotherapy. All participants will be followed up for at least 3 months and to the maximum time until study closure.

For peer review only

Trial intervention

Patients will receive radiotherapy online adaptive MRgRT on Viewray MRIdian MR Linac [20]. True Fast Imaging with Steady State Precession (TRUFISP) T2/T1weightedsequences are used to provide the cine MRI (real-time imaging) for beam gating, and volumetric imaging for OARs. SABR fractions will be delivered ideally on alternate days, treating at least twice per week. Daily treatment is permissible with at least 18 hours between fractions for five-fraction regimen.

Hypofractionation

For each of the three fractionations (50Gy in five fractions, 39Gy in three fractions or 25Gy in a single fraction) we have defined a single dose level (See Table 1). The starting regimen will be 50 Gy in 5 fractions (BED10 =100 Gy). Our in-silico planning study showed that three and single-fraction pancreas SABR plans could be generated while meeting organ dose constraints and delivering a meaningful dose to the target. Furthermore, treatments could be delivered within a reasonable timeframe [21].. Patients included in the study were from the CAP cohort (n=8, median GTV 41.35cc, (range 15.9-64.4). The median PTV V100 coverage for 39Gy in three fractions and 25Gy in one fraction was 75.7% (60.6-91.6%) and 66.1 (60.1-84.2%) respectively. The median treatment delivery times for 15.2min (12.5-21.7min) and 21.0min (15.9-33.2min) for 39Gy in three fractions and 25Gy in a single fraction respectively.

Table 1 – Table 1: Radiotherapy dose levels. BED 10 biological equivalent dose for acute reacting tissues (α/β=10).

Fractions	Dose to PTV (Gy)		
	Dose/#	Total dose	BED 10
5	10	50	100
3	13	39	90
1	25	25	88

Dose limiting toxicity (DLT)

In this study, DLT is defined in the following list of possible SABR treatment related adverse events (defined according to CTCAE v5.0) seen in the period from starting SABR treatment to three months post treatment.

DLT Events (within 3 months)

- Grade 3 upper gastro-intestinal (GI) bleeding
- GI fistula (any grade)
- Grade 4 nausea/vomiting uncontrolled despite optimum anti-emetics
- Grade 4 pancreatitis not stent related
- Vascular events (where these are not considered to be tumour related)

Late-onset severe toxicities

Late-onset severe toxicities may occur and will be monitored for during follow up (>3 months and up to 24 months where trial remains open and/or patient on trial)

- >=Grade 3 upper GI bleed
- gastro-intestinal fistula (any grade)
- >= Grade 3 vascular events

Trial population

For peer review only

The target population for the Emerald-Pancreas trial consists of male or female aged 16 years or above with an ECOG performance status of 0-1 with a localised pancreatic cancer. Patients are not permitted to enter the trial if they have a contraindication to having an MRI (presence of metallic implants, shrapnel, claustrophobia, or other expected intolerance of prolonged stay in an MRI scanner).

There are no specific restrictions on tumour size, number or interval from diagnosis. The following patients with localised pancreatic cancer may enter the trial: locally advanced and inoperable pancreatic cancer, inoperable on medical grounds, operable, but declines surgery and locally recurrent pancreatic cancer.

A full list of inclusion and exclusion criteria can be found in appendix 1. At least 3 months of chemotherapy prior to RT is recommended but not mandated. Chemotherapy should be avoided for at least 2 weeks before and for 4 weeks after RT.

Study objectives and end-points

The primary objective of the study is to establish the safety of five-, three- and single fraction MR-guided hypofractionation SABR in localised pancreatic cancer by assessing DLTs up to three months from start of radiotherapy. The secondary objectives include assessment of efficacy and long-term toxicity with the following endpoints: overall survival, progression free survival, local control rates, overall control rate, resection rates, long-term toxicities, and freedom from second line chemotherapy.

Planned Exploratory objectives for Emerald-Pancreas trial will include evaluation of imaging and immune biomarkers that may be useful to predict outcome from radiotherapy. Research blood samples will be collected baseline, 3 weeks and 3 months after the first RT fraction to monitor changes in immune cell markers in response to treatment in a cohort of up to 20 patients. The schedule for collection of data for the purposes of evaluating all trial endpoints is found in Appendix 2.

Radiotherapy planning

Patients will undergo a dedicated MR-Linac simulation as well as a CT simulation. CT simulation is to be used as primary data set for electron density information for planning dose calculation purposes. Alternatively, creation of synthetic CT simulation is acceptable where a suitable review of dosimetric accuracy using this approach has been carried out. Patient will be fasted four hours prior to scan and treatment. Intravenous contrast is required for the planning CT but not the planning MRI. Patient will be scanned in breath hold. A suitable tracking structure will be identified, either tumour in the first instance or suitable surrogate where tumour tracking is determined unsuitable by clinical team. Ability to track motion will be assessed through MR cine imaging.

The planning CT scan should ideally be carried out post MR simulation. Scan will be in the same breath hold used during MR simulation to maximise the chance of accurate deformation of the CT for use in planning calculations.

Delineation

Tumour volume definition is discussed and peer-reviewed with a pancreatic/upper GI radiologist and a second upper GI clinical oncologist. Outlining of the volumes of interest should take account of all available diagnostic imaging. Gross tumour volume (GTV) should not be reduced for a region that is negative on PET-CT but identified as abnormal on other imaging modalities. The following structures are to be created:

GTV_T = Macroscopic pancreatic tumour visible on imaging.

For peer review only

GTV_N = peritumoural lymph nodes >1 cm in short axis diameter and likely involved on diagnostic imaging. Include nodes < 1cm if considered involved by radiologist. Prophylactic nodal irradiation is not typically performed.

GTV_Final = GTV_T + GTV_N.

Clinical target volume (CTV) = GTV + 2 mm, with CTV cropped to the visceral OARs .

Planning target volume (PTV) = CTV + 3 mm.

The PTV will be divided into PTV_high and PTV_low so that any overlap of OAR and PTV still allows tolerance of OAR to be achieved. The overlap region needs to be designed and setup as rule so that it can be updated during plan adaptation. Nomenclature is as follows:

Critical structures (CS) = duodenum + small bowel + large bowel + stomach

PTV_high = PTV – (CS + 5 mm*)

PTV_low = intersection between PTV and (CS +5 mm*)

*A 5 - 8 mm margin may be needed from critical structures to achieve plan objectives.

Organs at risk defined below are to be contoured, plus any others considered relevant for the particular case as deemed by CI:

- Duodenum, stomach, small bowel, large bowel and oesophagus:
  Should be outlined on all slices from 3cm above to 3cm below the PTV. Individual loops of duodenum, small bowel and large bowel should be outlined.
- Kidneys
- Liver
- Spinal cord:
- Common bile duct, vessels and gallbladder are to be contoured when near to or overlapping the PTV to avoid hotspots.

Radiotherapy planning

Coplanar step-and-shoot IMRT plans are generated in a pseudo-arc formation. The planning objective is that 98% of PTV_high receives ≥95% of the prescribed dose. If the mandatory duodenum, bowel or stomach constraints cannot be met, PTV coverage should be reduced until the constraints are met. PTV_high coverage should not ideally need compromising, but coverage should also be reduced until mandatory constraints are met where needed. The minimum dose coverage objective of PTV V100%≥60%. The maximum dose in 1 cc of the PTV will be ≥125% and ≤ 140%. Hotspots should be within the GTV. OAR constraints are found in Table 2. All constraints are mandatory unless otherwise stated.

For peer review only

Table 2: Organs-at-risk dose constraints. All constraints are mandatory unless otherwise stated. (Vx denotes volume of organ receiving xGy, PRV – planning organ at risk volume, IVC – inferior vena cava)

**5 fractions**		
**Organ**	**Constraint**	**Volume**
Unified viscera constraint (Stomach Duodenum Small bowel Large bowel)	V36	0.1 cc
	V33	0.5 cc
	V25	20cc
Common bile duct	V50	0.5 cc
Liver	Mean	13 Gy
Kidney	Mean to either	10 Gy
Spinal cord PRV	V25<br>V14.5	£0.5 cc<br>£1 cc
Aorta/IVC	V53	0.5 cc
**3 fractions**		
Stomach Duodenum Small bowel Large bowel	V24<br>V22.2	0.5 cc (mandatory)<br>0.5 cc (optimal)
	V20<br>V16.5	5 cc (mandatory)<br>5 cc (optimal)
Common bile duct	V50	0.5 cc
Liver	V15	700 cc
Oesophagus	V25	0.5 cc
Kidneys	V16	200 cc
Spinal cord PRV	V18	0.1 cc
Aorta/IVC	V45	0.5 cc
**1 fraction**		
Duodenum Stomach Small bowel Large bowel	V12<br>V15	0.5 cc (optimal)<br>0.5 cc (mandatory)
	V11	5 cc
Common bile duct	V27	0.5cc
Liver	V9	700 cc
Oesophagus	V19	0.5 cc
Kidneys	V16	200 cc
Spinal cord PRV	V14	0.1 cc
Aorta/IVC	V37	0.5 cc

For peer review only

Treatment delivery

System integrated image registration between the simulation image dataset and the fraction image dataset will be performed. Original plan contours are propagated onto the respective fraction image dataset. All critical structures within a 3 cm axial and 2 cm craniocaudal distance from the surface of the original PTV will be re-contoured on the fraction image dataset. Tumour volume to be re-contoured at clinician discretion.

An estimated delivered dose will be calculated using the software on the console (dose prediction). Subsequently, an adapted radiotherapy plan is generated. During radiation dose delivery, continuous cine MR image acquisition in at least one principal plane (suggested sagittal, but at the discretion of the treating physician) is mandatory for soft tissue tracking and radiation beam gating. To this end, a

tracking slice will be positioned to include a cross-sectional cut of the target or suitable surrogate for intra- fractional soft tissue tracking. The tracking/gating volume will be delineated based on either the GTV or the PTV.

Breathing motion management will be employed. This will include shallow breathing and breath hold. Breath hold may be patient directed or based on staff coaching. Breath-hold assistance devices such as use of mirrors to visualize a wall mounted monitor, MR compatible goggles or image projection into the bore for target positional visualization are allowable and encouraged for use.

In patients receiving single-fraction, the fraction will be sub-divided into 2 pseudo-fractions to allow for a second IGRT match to be carried out. If this match is found to be acceptable and the patient is tolerating the treatment, the rest of the fraction will be delivered immediately. Otherwise, a second adaptive plan could be generated.

Pre-radiotherapy checks

Prospective contour and plan review will be done with an upper-GI radiologist and a second upper-GI site specialist. During treatment, attempts will be made to ensure a second MRL clinician is present. On treatment, secondary quality assurance (QA) contour check will be performed by a trained dosimetrist/medical physicist.

All complex plans must undergo patient specific pre-treatment or on-treatment QA according to local protocols with a fluence and point dose delivery check on the linac recommended using a suitable QA detector array device (e.g. ArcCheck). Pre-treatment QA measurements must use the baseline treatment plan. On-treatment QA should use the most recently delivered treatment fraction.

Analysis plan

A maximum of 60 evaluable patients will be included. There is no formal power calculation as this is a phase I trial, but simulations have been carried out to ensure the sample size is adequate. Conjugate posterior beta distributions will be used to evaluate the safety of five-, three- and single fraction using established dose constraints. The three fractionation regimens will be considered as three separate cohorts and analysed independently. The acceptable toxicity level is defined as 15% for each regimen. If the posterior probability of the DLT rate being above 15% is too high, (e.g. P(risk of DLT > 0.15 | Regimen, Data) > δ with δ defined using simulations before the start of the trial), that regimen will stop for safety. There is no early stopping for success.

A prior beta distribution will be specified for each regimen's toxicity rate. As binary (0=No DLT, 1=DLT) toxicity data is accrued in each regimen, these priors will be updated to conjugate posterior beta distributions. These represent the distribution for the probability of DLT rate within a specific regimen based on all available data. It is from these posterior distributions that inferences about safety will be made. The priors are calibrated to ensure the models provide sensible posterior probabilities based on prior clinical knowledge and incoming trial data.

Only patients for whom we have full information (e.g. experienced a DLT or completed DLT follow-up window) will be included in the modelling. Due to the low acceptable toxicity rate (15%) there is a concern a small number of DLTs early in the trial could result in the trial stopping for safety when in reality the treatment is safe. To reduce the possibility of erroneously stopping early, the model for each regimen will not run until 3 patients from that regimen have full toxicity information. If there are safety concerns prior to this, the trial management group may convene and decide the appropriate action.

Patient and Public Involvement

The Emerald trial has been reviewed and endorsed by patient and carer representatives. Patient and public involvement (PPI) began at the protocol design and development stage. This included a review of the trial patient information sheet and consent form. Further consumer feedback was obtained at the CTRad proposal guidance meeting. Their feedback was adopted and incorporated into the final version of the patient information sheet. PPI representatives will also be invited to attend trial management group meetings throughout the trial.

Author contributions

For peer review only

All contributors meet at least one of the criteria recommended by the ICMJE. ST, TM and SM conceived the study design. ST, AO, BG, KYC, JD, MP, LG, JG, TS and SM were involved in protocol development and manuscript.

Acknowledgments: The trial is coordinated by the Oncology Clinical Trials Office (OCTO) at the University of Oxford with statistical support from the Centre for Statistics in Medicine, which together form part of the Oxford Clinical Trials Research Unit (OCTRU), a UKCRC Registered Clinical Trials Unit.

Funding: This work is funded through the Oxford University-Genesis Care Collaboration Fund (award/grant number: n/a), the John Black Charitable Foundation (award/grant number: n/a) and Oxford Institute for Radiation Oncology, University of Oxford (award/grant number: n/a). ST is Clinical Career Development Fellow funded by CRUK (award:ART-Net grant). SM is part-funded by Biomedical Research Oxford (award/grant number: n/a). Standard treatment costs for radiotherapy is funded by the NHS (award/grant number: n/a).

Sponsor: The trial is sponsored by the University of Oxford.

Trial registration: ISRCTN10557832

Competing Interest: We declare none of the authors have competing interest.

For peer review only

References

1. Iacobuzio-Donahue CA, Fu B, Yachida S, et al. DPC4 gene status of the primary carcinoma correlates with patterns of failure in patients with pancreatic cancer. *J Clin Oncol* 2009;27(11):1806-13. doi: 10.1200/JCO.2008.17.7188 [published Online First: 20090309]
2. Chuong MD, Springett GM, Freilich JM, et al. Stereotactic body radiation therapy for locally advanced and borderline resectable pancreatic cancer is effective and well tolerated. *Int J Radiat Oncol Biol Phys* 2013;86(3):516-22. doi: 10.1016/j.ijrobp.2013.02.022 [published Online First: 20130405]
3. Mahadevan A, Miksad R, Goldstein M, et al. Induction gemcitabine and stereotactic body radiotherapy for locally advanced nonmetastatic pancreas cancer. *Int J Radiat Oncol Biol Phys* 2011;81(4):e615-22. doi: 10.1016/j.ijrobp.2011.04.045 [published Online First: 20110612]
4. Chang DT, Schellenberg D, Shen J, et al. Stereotactic radiotherapy for unresectable adenocarcinoma of the pancreas. *Cancer* 2009;115(3):665-72. doi: 10.1002/cncr.24059
5. Didolkar MS, Coleman CW, Brenner MJ, et al. Image-guided stereotactic radiosurgery for locally advanced pancreatic adenocarcinoma results of first 85 patients. *J Gastrointest Surg* 2010;14(10):1547-59. doi: 10.1007/s11605-010-1323-7 [published Online First: 20100914]
6. Liauw SL, Ni L, Wu T, et al. A prospective trial of stereotactic body radiation therapy for unresectable pancreatic cancer testing ablative doses. *J Gastrointest Oncol* 2020;11(6):1399-407. doi: 10.21037/jgo-20-187 [published Online First: 2021/01/19]
7. Koong AC, Le QT, Ho A, et al. Phase I study of stereotactic radiosurgery in patients with locally advanced pancreatic cancer. *Int J Radiat Oncol Biol Phys* 2004;58(4):1017-21. doi: 10.1016/j.ijrobp.2003.11.004
8. Schellenberg D, Goodman KA, Lee F, et al. Gemcitabine chemotherapy and single-fraction stereotactic body radiotherapy for locally advanced pancreatic cancer. *Int J Radiat Oncol Biol Phys* 2008;72(3):678-86. doi: 10.1016/j.ijrobp.2008.01.051 [published Online First: 20080418]
9. Hammel P, Huguet F, van Laethem JL, et al. Effect of Chemoradiotherapy vs Chemotherapy on Survival in Patients With Locally Advanced Pancreatic Cancer Controlled After 4 Months of Gemcitabine With or Without Erlotinib: The LAP07 Randomized Clinical Trial. *JAMA* 2016;315(17):1844-53. doi: 10.1001/jama.2016.4324
10. Hurt CN, Falk S, Crosby T, et al. Long-term results and recurrence patterns from SCALOP: a phase II randomised trial of gemcitabine- or capecitabine-based chemoradiation for locally advanced pancreatic cancer. *Br J Cancer* 2017;116(10):1264-70. doi: 10.1038/bjc.2017.95 [published Online First: 20170404]
11. Hoyer M, Roed H, Sengelov L, et al. Phase-II study on stereotactic radiotherapy of locally advanced pancreatic carcinoma. *Radiother Oncol* 2005;76(1):48-53. doi: 10.1016/j.radonc.2004.12.022
12. Schellenberg D, Kim J, Christman-Skieller C, et al. Single-fraction stereotactic body radiation therapy and sequential gemcitabine for the treatment of locally advanced pancreatic cancer. *Int J Radiat Oncol Biol Phys* 2011;81(1):181-8. doi: 10.1016/j.ijrobp.2010.05.006 [published Online First: 20110505]
13. Good J, George B, Teoh S, et al. OC-0112 Feasibility and safety of daily adapted MR-guided SABR for pancreatic cancer in the UK. *Radiotherapy and Oncology* 2022;170:S86-S87. doi: 10.1016/S0167-8140(22)02488-4

For peer review only

14. Rudra S, Jiang N, Rosenberg SA, et al. Using adaptive magnetic resonance image-guided radiation therapy for treatment of inoperable pancreatic cancer. *Cancer Med* 2019;8(5):2123-32. doi: 10.1002/cam4.2100 [published Online First: 20190401]
15. Hall WA, Small C, Paulson E, et al. Magnetic Resonance Guided Radiation Therapy for Pancreatic Adenocarcinoma, Advantages, Challenges, Current Approaches, and Future Directions. *Front Oncol* 2021;11:628155. doi: 10.3389/fonc.2021.628155 [published Online First: 20210511]
16. M. Chuong; K. Mittauer; R. Herrera; T. Romaguera; D. Alvarez; R. Kotecha; M. Hall AKJBJMA. OC-0415 Long-term outcomes of MR-guided SABR & on-table adaptive replanning for unresectable pancreas cancer. *Radiotherapy and Oncology* 2021;161:S310-S11. doi: https://doi.org/10.1016/S0167-8140(21)06902-4.
17. Sivakumar S, Abu-Shah E, Ahern DJ, et al. Activated Regulatory T-Cells, Dysfunctional and Senescent T-Cells Hinder the Immunity in Pancreatic Cancer. *Cancers (Basel)* 2021;13(8) doi: 10.3390/cancers13081776 [published Online First: 20210408]
18. Ye J, Mills BN, Zhao T, et al. Assessing the Magnitude of Immunogenic Cell Death Following Chemotherapy and Irradiation Reveals a New Strategy to Treat Pancreatic Cancer. *Cancer Immunol Res* 2020;8(1):94-107. doi: 10.1158/2326-6066.CIR-19-0373 [published Online First: 20191112]
19. Mills BN, Qiu H, Drage MG, et al. Modulation of the Human Pancreatic Ductal Adenocarcinoma Immune Microenvironment by Stereotactic Body Radiotherapy. *Clin Cancer Res* 2022;28(1):150-62. doi: 10.1158/1078-0432.CCR-21-2495 [published Online First: 20211203]
20. Kluter S. Technical design and concept of a 0.35 T MR-Linac. *Clin Transl Radiat Oncol* 2019;18:98-101. doi: 10.1016/j.ctro.2019.04.007 [published Online First: 20190408]
21. Chu K, Teoh S, Maughan T, et al. PO-1677 Planning feasibility study of three and single fraction Pancreas MR-Linac SBRT - Phase 1 trial setup. *Radiotherapy and Oncology* 2022;170:S1476-S77. doi: 10.1016/S0167-8140(22)03641-6

For peer review only

Figure 1 – Trial recruitment phases. (DLT – dose limiting toxicities).

Figure 2 - Trial schema and overview of follow-up (MDT- multidisciplinary team)

For peer review only

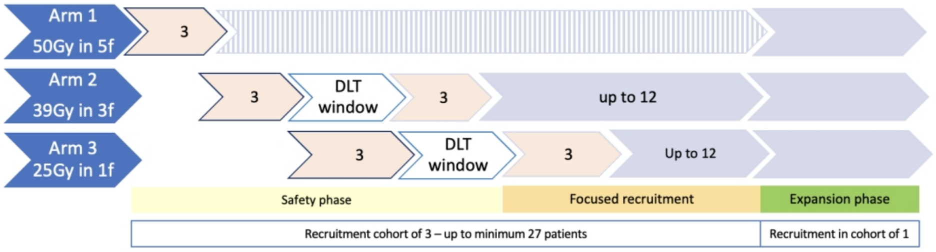

Figure 1 – Trial recruitment phases. (DLT – dose limiting toxicities).

285x83mm (144 x 144 DPI)

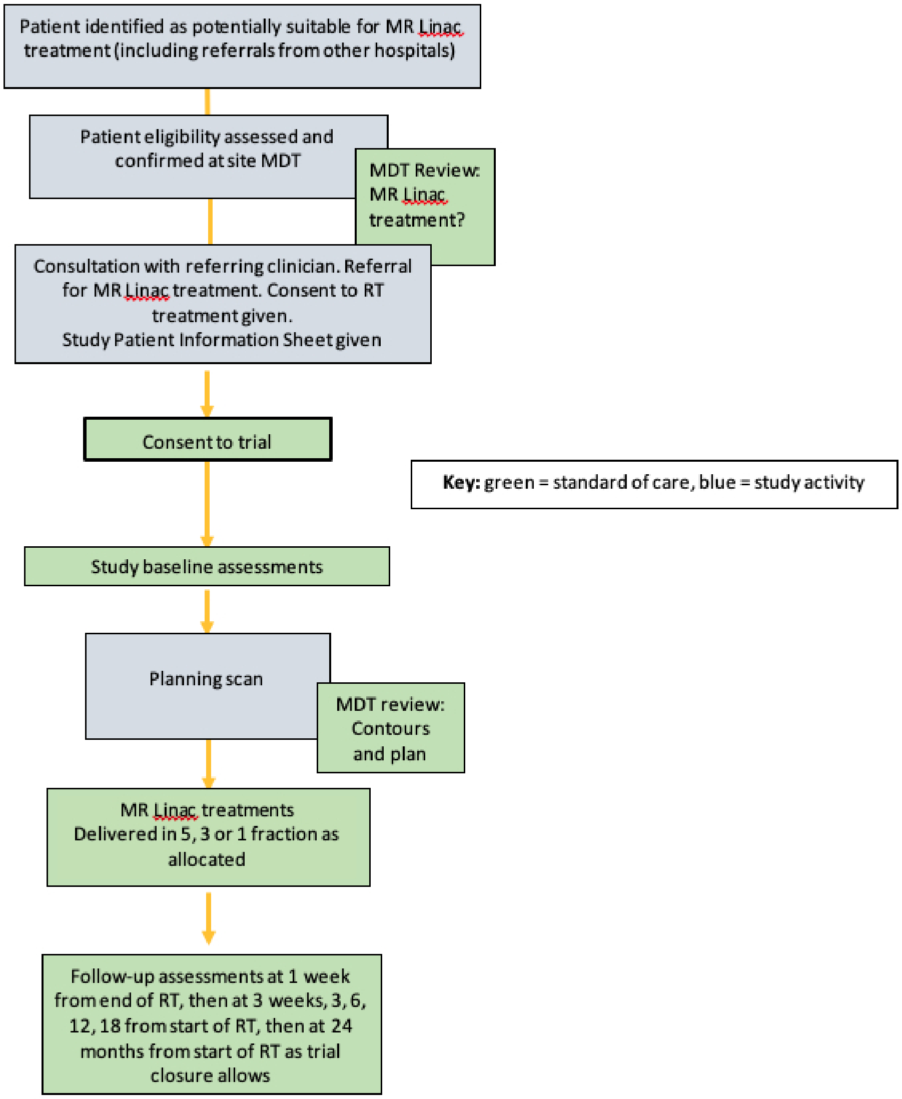

Figure 2 - Trial schema and overview of follow-up (MDT- multidisciplinary team)

213x264mm (72 x 72 DPI)

Eligibility criteria for entry into the main study

Inclusion criteria:

A patient will be eligible for inclusion in this study if all of the following criteria apply.

1. Participants must be fit and scheduled to receive MR guided radiotherapy for pancreatic cancer. There are no specific restrictions on tumour size, number or interval from diagnosis.
2. Localised pancreatic cancer, which may be
    a. locally advanced and inoperable pancreatic cancer
    b. inoperable on medical grounds
    c. operable, but patient declines surgery
    d. locally recurrent pancreatic cancer

3. Histologically proven pancreatic ductal adenocarcinoma or cytological proven pancreatic malignancy. Where histology/cytology is 'suspicious' MDT should confirm that it is appropriate to treat as malignancy.
4. Male or Female, aged 16 years or above.
5. Life expectancy of at least 6 months.
6. ECOG performance status 0- 1.
7. Haematological and biochemical indices within defined ranges:

Lab Test	Value required
Haemoglobin (Hb)	≥ 8.0 g/dL
Platelet count	$\geq 50 \times 10^9$/l;
Neutrophils	$\geq 1.0 \times 10^9$/l.
Total bilirubin	≤ 1.5 x IULN
AST(SGOT) or ALT(SGPT)	≤ 3.0 x IULN

8. Able (in the investigators' opinion) and willing to comply with all study requirements for the duration of the study.
9. Willing and able to give informed consent.

Exclusion criteria:

A patient will not be eligible for the trial if any of the following apply:

1. Patients with specific MRI exclusion criteria – metallic implants, shrapnel, claustrophobia or other expected intolerance of prolonged (up to 90 minutes) stay in MRI scanner.
2. Prior radiotherapy to the upper abdomen
3. Pregnant or breast-feeding women, or women of childbearing potential unless effective methods of contraception are used. Male patients who do not agree to use a condom during RT treatment and for three months after or who are not surgically sterile.
4. Distant metastatic disease or local disease that cannot be encompassed in the SABR field.

For peer review only

SCHEDULE OF ASSESSMENTS

Procedure *(grey shading denotes SoC activities)*	Pre-screening Within 3 months of RT #1	Screening & baseline Within 28 Days of planning scan	Planning scan visit	RT fraction[14]					Post radiotherapy							Progression (if applicable)	Early Withdrawal (if applicable)	End of study[19] (+/-1 months)
				#1	#2	#3	#4	#5	+1 weeks[16] (+/- 4 days)	3 weeks[17] (+/- 1 wk)	6 weeks[17] (+/- 2 wks)	3 months[17] (+2 wks)	6 months[17] (+/- 1 mon)	12 months[17] (+/- 1 mon)	18 months[17] (+/- 1 mon)			
CT Scan TAP [1]	X											X	X	X		X		
Informed consent [2]		X																
Demographics[3]		X																
Baseline sign & symptoms[4]		X																
Medical history		X																
Haematology[5]		X								X		X						
Biochemistry[6,7]		X								X		X						
Ca19.9 tumour marker		X								X		X						
Urine pregnancy test (WOCBP only)		X																
Clinical review & disease assessment [8]		X							X	X	X	X	X	X	X		X	
Performance status		X		X[15]					X		X	X	X	X	X			
AE review [9]			X[21]	X	X	X	X	X	X	X	X	X	X	X	X		X	X
DLT review				X	X	X	X	X	X	X	X	X					X[18]	
Late-onset severe toxicities review													X	X	X		X[20]	X
Planning scan (0.35T MRI)			X															
MR Linac treatment [11]				X	X	X	X	X										
Optional research blood sample[12]		X								X		X						
Data capture: PFS, survival status, resection status, restart of chemotherapy & locoregional failure[13]												X	X	X [19]	X	X	X [19]	X [19]
Reason for withdrawal																	X	

1. Standard of care (SOC) diagnostic CT and optional MRI are used as baseline imaging and whole-body PET-CT if performed in routine care. Further CT scans (& MRI, PET-CT where applicable) at 3, 6 & 12 months follow-up and if relapse/progression occurs are also standard of care. Scans and clinical report to be pseudonymised with trial subject ID and transferred to OU. Scan images and report need to be transferred to the OU if scan carried out at another trust.
2. See section 4.5 for further information on informed consent process.
3. Demographic details to include age and sex
4. Baseline Sign and Symptoms: provide date of onset, event diagnosis (if known) or sign/symptom, severity, time course. Terms should be specific medical terms according to NCI CTCAE version 5. Please avoid using abbreviations, combined terms e.g. nausea and vomiting and ambiguous terms e.g. deranged, abnormal.
5. Haematology: Full Blood Count.
6. eGFR at baseline only.
7. Biochemistry: sodium, potassium, urea and electrolytes, creatinine, ALT or AST, Bilirubin, Albumin, alkaline phosphatase.
8. As considered appropriate by clinician. In follow-up this should include disease assessment for progression
9. AE assessment may be undertaken face to face, by telephone or audio/video call through the internet.
10. The planning scan is required as a routine part of clinical care, for the study an additional set of research images may be acquired during the planning scan.
11. MR Linac treatment will be given in 5, 3 or 1 fractions as per dose selection process detailed in section 10.0 & 10.1.
12. Optional Research Blood Sample: For patients who have consented: Research bloods to be taken pre-treatment, 3 week and 3 months post RT.
13. For participants who have not reached 24 months follow-up at study closure this data will be collected at an earlier timepoint, at least 3 months after start of RT and as close as possible to study closure.
14. Fractions may be missed/delayed at Investigators' discretion
15. ECOG status prior to fraction 1.
16. From the end of radiotherapy
17. From start of radiotherapy
18. If early withdrawal is within 3-month DLT collection window
19. Evaluations to be completed at end of study, 24 months (from RT Fraction #1) follow-up or death
20. If early withdrawal is after 3-month DLT Collection window.
21. The AE Review at the Planning Scan Visit can be performed with the RT Fraction #1 AE review, as long as the review includes AE assessment from the Planning Scan Visit date.

For peer review only

BMJ Open

Evaluation of hypofractionated adaptive radiotherapy using the MR Linac in localised pancreatic cancer (Emerald-Pancreas): Phase 1/expansion study

Journal:	*BMJ Open*
Manuscript ID	bmjopen-2022-068906.R1
Article Type:	Protocol
Date Submitted by the Author:	08-Jul-2023

Complete List of Authors:	Teoh, Suliana; University of Oxford Department of Oncology; Oxford University Hospitals NHS Foundation Trust<br>Ooms, Alexander; University of Oxford, Nuffield Department of Orthopaedics, Rheumatology and Musculoskeletal Disorders<br>George, Ben; GenesisCare UK<br>Owens, Rob; Oxford University Hospitals NHS Foundation Trust; GenesisCare UK<br>Chu, Kwun-Ye; University of Oxford Department of Oncology; Oxford University Hospitals NHS Foundation Trust<br>Drabble, Joe; GenesisCare UK<br>Robinson, Maxwell; Oxford University Hospitals NHS Foundation Trust<br>Parkes, Matthew; Oxford University, Oxford Clinical Trials Research Unit (OCTRU)<br>Swan, Lynda; University of Oxford Department of Oncology<br>Griffiths, Lucinda; University of Oxford Department of Oncology<br>Nugent, Killian; Oxford University Hospitals NHS Foundation Trust, Department of Oncology; GenesisCare UK, Oncology<br>Good, James; GenesisCare UK<br>Maughan, Tim; University of Oxford Department of Oncology; Oxford University Hospitals NHS Foundation Trust<br>Mukherjee, Somnath; University of Oxford Department of Oncology; Oxford University Hospitals NHS Foundation Trust

**Primary Subject Heading**:	Oncology
Secondary Subject Heading:	Gastroenterology and hepatology
Keywords:	Magnetic resonance imaging < RADIOTHERAPY, Hepatobiliary tumours < ONCOLOGY, Radiation oncology < RADIOTHERAPY

SCHOLARONE™
Manuscripts

Title: Evaluation of hypofractionated adaptive radiotherapy using the MR Linac in localised pancreatic cancer (Emerald-Pancreas): Phase 1/expansion study

Authors: S Teoh[1,2], A Ooms[3], B George[4], R Owens[2,3], KY Chu[1,2], J Drabble[4], M Robinson[2], M Parkes[3], L Swan[1], L Griffiths[1], K Nugent[1,4], J Good[4], T Maughan[1], S Mukherjee[1,2]

[1] Department of Oncology, University of Oxford, OX3 7DQ, UK
[2] Oxford University Hospitals NHS Foundation Trust, OX3 7LE, UK
[3] Nuffield Department of Orthopaedics, Rheumatology and Musculoskeletal Disorders, University of Oxford, OX3 7LD, UK
[4] GenesisCare UK, OX4 6LB, UK

Corresponding author: Prof Somnath Mukherjee

Corresponding address:
Department of Oncology
University of Oxford
Old Road Campus Research Building
Roosevelt Drive
Oxford OX3 7DQ

Mobile: +44 7921855863

Email: Somnath.mukherjee@ouh.nhs.uk

Keywords: Pancreatic cancer, online adaptive radiotherapy, MR-guided radiotherapy, SABR

Word count: 3925

For peer review only

Abstract

Introduction:
Online adaptive magnetic resonance guided radiotherapy (MRgRT) allows for dose escalation to pancreatic cancer while sparing surrounding critical organs. We seek to evaluate the safety of delivering hypofractionated five-, three- and single fraction MR-guided stereotactic ablative radiotherapy (SABR) to the pancreas.

Methods and analysis:
This is a single centre three-arm phase 1 non-randomised safety study. Patients with localised pancreatic cancer will receive either 50Gy in five- (BED_{10} =100Gy), 39Gy in three- (BED_{10} =90Gy), or 25Gy in a single fraction (BED_{10} =87.5Gy) MR-guided daily online adaptive radiotherapy. Each fractionation regime will be assessed as independent cohorts to determine tolerability, assessed continuously using Bayesian conjugate posterior beta distributions. The primary endpoint of the study is to establish the safety of five-, three- and single fraction MR-guided hypofractionation SABR in localised pancreatic cancer by assessing dose-limiting toxicities. Secondary endpoints include overall survival, progression free survival, local control rates, overall control rate, resection rates, long-term toxicities and freedom from second line chemotherapy. This study plans to also explore imaging and immune biomarkers that may be useful to predict outcome and personalise treatment. The trial will recruit up to 60 patients with a safety run-in.

Ethics and dissemination: The trial is approved by the West Midlands - Black Country Research Ethics Committee 22/WM/0122.The results will be disseminated via conference presentations, peer-reviewed scientific journals and submission to regulatory authorities.

Trial registration number: ISRCTN10557832

Keywords:
Pancreatic cancer, online adaptive radiotherapy, MR-guided radiotherapy, SABR

Strength and Limitations

- This is a phase I safety study evaluating online adaptive extreme hypofractionated radiotherapy using novel MR-guided-radiotherapy (strength).
- There is continuous monitoring of the primary outcome of dose limiting toxicity using conjugate posterior beta distribution (strength).
- Longer term outcome data is limited due to follow-up period of up to 24 months (limitation).
- The trial has a non-comparative design (limitation).
- All three fractionation regimes will be considered as three separate cohorts and therefore outcome could be that three different SABR prescriptions are safe to deliver in pancreatic cancer (strength).

For peer review only

Background

The role of radiotherapy in patients with inoperable pancreatic cancer is controversial due to its high propensity to metastasize. Meaningful benefit of local control is challenging to quantify when most patients die of distant metastatic disease. Nevertheless, not all patients with adenocarcinoma of the pancreas develop distant metastatic disease. A third of these patients die mainly of local progression [1]. As systemic treatment improves, local control is likely to be increasingly important. Stereotactic ablative radiotherapy (SABR) where an ablative dose of RT is delivered to a small volume in one to five fractions, has been shown to achieve local control rates as high as 80-100%[2-8], compared to about 50-70% reported in CRT trials [9 10]. Initial SABR experiences for treatment of locally advanced pancreatic cancer with ablative doses of radiation had reported high rates of toxicity, particularly with regimens delivered in less than 5 fractions [5 6 11].

There are a number of challenges in delivering highly conformal radiotherapy in pancreatic cancer. Firstly, tumour and organs-at-risk (OAR) visualisation during treatment setup is limited with CT-based imaging. Moreover, the proximity of pancreatic tumours to highly mobile radiosensitive organs such as the duodenum, stomach and bowel means delivering high doses while sparing these organs is difficult. A number of studies have explored the use of SABR to improve outcome. Of concern is the toxicity rate observed in some of these studies. Hoyer et al treated patients with 45Gy in 3 fractions and reported 5 out of 22 patients developing severe mucositis or perforation[11]. Not all patients had on-board CT-imaging for radiotherapy setup. Similarly, Liauw et al reported up to 27% grade 3 and above late toxicities following three-fraction SABR [6]. In both cases large PTV margin and no motion management was utilised. Schellenberg et al evaluated concurrent gemcitabine with single fraction 25 Gy SABR for LAPC and reported freedom from local progression was 88% at 1 year. However, they reported 15% grade 2 ulcers and one patient with a duodenal perforation [12].

Daily online adaptive magnetic resonance imaging (MRI)-guided radiotherapy (MRgRT) is a novel method of delivering radiotherapy that could potentially allow for dose escalation without exceeding dose to the organs-at-risk (OAR). MRI provides superior visualisation of organs such as the stomach, duodenum, and bowel as well as improved tumour identification. Online adaptive MRgRT gives the opportunity to adapt treatment plans daily to account for inter-fraction variability of these organs to maintain target coverage while sparing high doses to OAR [13], Teoh, et al ASTRO 22 poster). Furthermore, the ability to track the tumour during treatment delivery means 'real-time' intra-fraction monitoring is possible.

Early data from a retrospective study shows that dose escalation to 50Gy in 5 fractions using online adaptive MRgRT is safe and may result in improved survival [14]. This retrospective analysis of 42 locally advanced pancreatic cancer patients treated by MR-guided adaptive RT at four institutions demonstrated that high-dose SABR or hypofractionated radiation therapy delivered using daily adaptive dose planning has the potential to further improve overall survival [14] . A control group of 19 patients treated to more conventional radiation doses without frequent dose adaptation showed a median survival of 14.8 months, while patients treated to high radiation doses (n=23, maximum BED10 of >90 Gy) under daily or almost daily adaptive re-planning had an estimated median survival of 27.8 months (p=0.005). Increased radiation dose delivery using daily dose adaptation was correlated with less grade 3 toxicity (0% in the high dose group vs 15.8% in patients treated to lower radiation doses without dose adaptation). Hall et al, summarised the current published experience for MRgRT in pancreatic cancer and found local control rates of between 77-88% at 1 year with toxicity rates of less than 10% (n=141 from 6 studies mostly retrospective) [15]. Chuong et al, retrospectively analysed 50 pancreatic cancer patients treated with 50Gy in 5 fraction using online daily adaptation on the ViewRay MRlinac and found one-year, and 2-year estimated local control were 97.8%, and 88.9%, respectively. Median survival was 21 months (1 and 2-year OS 87.9% and 50%) (ESTRO 2021) [16].

Pancreatic adenocarcinoma features both local and systemic immunosuppression[17]. This enables immune escape and establishment of the tumour both at the primary and distant sites. Whether or not ablative radiation doses to the primary tumour can induce systemic tumour immunity in pancreatic cancer is unknown. In murine pancreatic ductal adenocarcinoma (PDAC) models, SABR has been shown to induce immunogenic cell death (ICD) and promote tumour cell antigen presentation resulting

For peer review only

in activation of tumour-specific T cells [18]. Mills et al evaluated the immune response from histopathological samples of patients treated with SABR (25Gy in 5 fractions) pre-operatively vs surgery alone and found that SABR reduced PDAC cell density, induced ICD and increased the proportion of PD-1+ T cells. However, despite these finding, intra-tumour T cell levels remain low and outnumbered by myeloid suppressors[19]. While SABR to an unselected population of LAPC patients is unlikely to significantly impact survival outcomes, there is potential for a multi-modality approach with immunotherapy, particularly when combined with biomarkers suggestive of a locally aggressive rather than metastatic phenotype.

The Emerald-Pancreas trial will evaluate the safety of delivering five-, three and single fractions SABR to the pancreas using online adaptive MRgRT. Shorter fractionation schedules are more convenient for patients and increase access to and cost-effectiveness of the scarce and expensive resource of an MRgRT machines. Being able to achieve high rates of local control of the primary tumour with minimal side effects and limited impact on patient lives would be a great advantage for patients. It will also enable greater access for systemic therapies, including conventional chemotherapy and novel immunotherapies, to develop more effective long term control regimens to combat this highly aggressive disease. The trial is currently actively recruiting with the first patient first visit (FPFV) being on 25th of August 2022 with the end of study date being the 31st of December 2024. Our last patient first visit (LPFV) is planned for the 30th of June 2024.

Methods/Design

The aim of the trial is to assess safety of extreme hypofractionation of SABR using MRgRT in pancreatic cancer. The five-, three- and single fraction MRgRT treatments will be assessed as independent cohorts to determine if each one is tolerable. Tolerability will be assessed using conjugate posterior beta distributions.

Trial design

This is a three-arm, non-randomised, open, safety study. There are three phases of recruitment to the study: an initial safety run-in, a focussed recruitment phase, and an expansion phase (see Figure 1).

In the initial safety run-in, three patients will be recruited into the five-fraction regimen. The three-fraction regimen will open immediately once three patients have been recruited into five-fraction regimen. Once three patients have been accrued to the three-fraction regimen, the single fraction regimen can open immediately. Recruitment to the one- and three-fractions regimens will pause while waiting for 3 months dose limiting toxicity (DLT) follow-up from the first three patients in each cohort. It is possible both the single and three fraction regimens will be paused at the same time. Any patients recruited whilst both one- and three-fractions regimens are paused will be assigned the five-fraction regimen.

Following the safety run-in, the focussed recruitment will start. Recruitment will continue in cohorts of 3 alternating between the three- and one-fraction regimens until a total of 12 patients in the one- and three-fraction regimens have been recruited. Recruitment to this phase will be continuous. After 3 patients' full DLT follow-up data in any regimen, the beta binomial model for that regimen can be run to assess the safety stopping rule in the event of a DLT.

For the remainder of the trial, the expansion phase recruitment will be in cohorts of 1 to all three regimens and recruitment will be continuous, alternating between one-, three-, and five-fractions. Where plans that meet dose constraints are unable to be generated for single fraction regimens that patient will be assigned to the three-fraction regimen or alternatively the five-fraction regimen if recruitment to the three-fraction cohort is paused or closed. Where plans that meet dose constraints are unable to be generated for three-fraction regimens, that patient will be assigned to the five-fraction regimen. These patients will be included in the main analysis for the regimen they are treated in. A sensitivity analysis will be performed excluding these patients as they may be at higher risk of toxicity. Figure 2 shows the trial schema and overview of follow-up. The duration of the study will be up to 24

For peer review only

months from patient starting radiotherapy. All participants will be followed up for at least 3 months and to the maximum time until study closure.

Trial intervention

Patients will receive radiotherapy online adaptive MRgRT on Viewray MRIdian MR Linac [20]. True Fast Imaging with Steady State Precession (TRUFISP) T2/T1weightedsequences are used to provide the cine MRI (real-time imaging) for beam gating, and volumetric imaging for OARs. SABR fractions will be delivered ideally on alternate days, treating at least twice per week. Daily treatment is permissible with at least 18 hours between fractions for five-fraction regimen.

Hypofractionation

For each of the three fractionations (50Gy in five fractions, 39Gy in three fractions or 25Gy in a single fraction) we have defined a single dose level (See Table 1). The starting regimen will be 50 Gy in 5 fractions (BED10 =100 Gy). Our in-silico planning study showed that three and single-fraction pancreas SABR plans could be generated while meeting organ dose constraints and delivering a meaningful dose to the target. Furthermore, treatments could be delivered within a reasonable timeframe [21].. Patients included in the study were from the CAP cohort (n=8, median GTV 41.35cc, (range 15.9-64.4). The median PTV V100 coverage for 39Gy in three fractions and 25Gy in one fraction was 75.7% (60.6-91.6%) and 66.1 (60.1-84.2%) respectively. The median treatment delivery times for 15.2min (12.5-21.7min) and 21.0min (15.9-33.2min) for 39Gy in three fractions and 25Gy in a single fraction respectively.

Table 1 – Table 1: Radiotherapy dose levels. BED 10 biological equivalent dose for acute reacting tissues (α/β=10).

Fractions	Dose to PTV (Gy)		
	Dose/#	Total dose	BED 10
5	10	50	100
3	13	39	90
1	25	25	88

Dose limiting toxicity (DLT)

In this study, DLT is defined in the following list of possible SABR treatment related adverse events (defined according to CTCAE v5.0) seen in the period from starting SABR treatment to three months post treatment.

DLT Events (within 3 months)

- Grade 3 upper gastro-intestinal (GI) bleeding
- GI fistula (any grade)
- Grade 4 nausea/vomiting uncontrolled despite optimum anti-emetics
- Grade 4 pancreatitis not stent related
- Vascular events (where these are not considered to be tumour related)

Late-onset severe toxicities

Late-onset severe toxicities may occur and will be monitored for during follow up (>3 months and up to 24 months where trial remains open and/or patient on trial)

- >=Grade 3 upper GI bleed
- gastro-intestinal fistula (any grade)
- >= Grade 3 vascular events

For peer review only

Trial population

The target population for the Emerald-Pancreas trial consists of male or female aged 16 years or above with an ECOG performance status of 0-1 with a localised pancreatic cancer. Patients are not permitted to enter the trial if they have a contraindication to having an MRI (presence of metallic implants, shrapnel, claustrophobia, or other expected intolerance of prolonged stay in an MRI scanner).

There are no specific restrictions on tumour size, number or interval from diagnosis. The following patients with localised pancreatic cancer may enter the trial: locally advanced and inoperable pancreatic cancer, inoperable on medical grounds, operable, but declines surgery and locally recurrent pancreatic cancer.

A full list of inclusion and exclusion criteria can be found in appendix 1. At least 3 months of chemotherapy prior to RT is recommended but not mandated. Chemotherapy should be avoided for at least 2 weeks before and for 4 weeks after RT.

Study objectives and end-points

The primary objective of the study is to establish the safety of five-, three- and single fraction MR-guided hypofractionation SABR in localised pancreatic cancer by assessing DLTs up to three months from start of radiotherapy. The secondary objectives include assessment of efficacy and long-term toxicity with the following endpoints: overall survival, progression free survival, local control rates, overall control rate, resection rates, long-term toxicities, and freedom from second line chemotherapy.

Planned Exploratory objectives for Emerald-Pancreas trial will include evaluation of imaging and immune biomarkers that may be useful to predict outcome from radiotherapy. Research blood samples will be collected baseline, 3 weeks and 3 months after the first RT fraction to monitor changes in immune cell markers in response to treatment in a cohort of up to 20 patients. The schedule for collection of data for the purposes of evaluating all trial endpoints is found in Appendix 2.

Radiotherapy planning

Patients will undergo a dedicated MR-Linac simulation as well as a CT simulation. CT simulation is to be used as primary data set for electron density information for planning dose calculation purposes. Alternatively, creation of synthetic CT simulation is acceptable where a suitable review of dosimetric accuracy using this approach has been carried out. Patient will be fasted four hours prior to scan and treatment. Intravenous contrast is required for the planning CT but not the planning MRI. Patient will be scanned in breath hold. A suitable tracking structure will be identified, either tumour in the first instance or suitable surrogate where tumour tracking is determined unsuitable by clinical team. Ability to track motion will be assessed through MR cine imaging.

The planning CT scan should ideally be carried out post MR simulation. Scan will be in the same breath hold used during MR simulation to maximise the chance of accurate deformation of the CT for use in planning calculations.

Delineation

Tumour volume definition is discussed and peer-reviewed with a pancreatic/upper GI radiologist and a second upper GI clinical oncologist. Outlining of the volumes of interest should take account of all available diagnostic imaging. Gross tumour volume (GTV) should not be reduced for a region that is negative on PET-CT but identified as abnormal on other imaging modalities. The following structures are to be created:

GTV_T = Macroscopic pancreatic tumour visible on imaging.

For peer review only

GTV_N = peritumoural lymph nodes >1 cm in short axis diameter and likely involved on diagnostic imaging. Include nodes < 1cm if considered involved by radiologist. Prophylactic nodal irradiation is not typically performed.

GTV_Final = GTV_T + GTV_N.

Clinical target volume (CTV) = GTV + 2 mm, with CTV cropped to the visceral OARs .

Planning target volume (PTV) = CTV + 3 mm.

The PTV will be divided into PTV_high and PTV_low so that any overlap of OAR and PTV still allows tolerance of OAR to be achieved. The overlap region needs to be designed and setup as rule so that it can be updated during plan adaptation. Nomenclature is as follows:

Critical structures (CS) = duodenum + small bowel + large bowel + stomach

PTV_high = PTV – (CS + 5 mm*)

PTV_low = intersection between PTV and (CS +5 mm*)

*A 5 - 8 mm margin may be needed from critical structures to achieve plan objectives.

Organs at risk defined below are to be contoured, plus any others considered relevant for the particular case as deemed by CI:

- Duodenum, stomach, small bowel, large bowel and oesophagus:
  Should be outlined on all slices from 3cm above to 3cm below the PTV. Individual loops of duodenum, small bowel and large bowel should be outlined.
- Kidneys
- Liver
- Spinal cord:
- Common bile duct, vessels and gallbladder are to be contoured when near to or overlapping the PTV to avoid hotspots.

Radiotherapy planning

Coplanar step-and-shoot IMRT plans are generated in a pseudo-arc formation. The planning objective is that 98% of PTV_high receives ≥95% of the prescribed dose. If the mandatory duodenum, bowel or stomach constraints cannot be met, PTV coverage should be reduced until the constraints are met. PTV_high coverage should not ideally need compromising, but coverage should also be reduced until mandatory constraints are met where needed. The minimum dose coverage objective of PTV V100%≥60%. The maximum dose in 1 cc of the PTV will be ≥125% and ≤ 140%. Hotspots should be within the GTV. OAR constraints are found in Table 2. All constraints are mandatory unless otherwise stated.

Table 2: Organs-at-risk dose constraints. All constraints are mandatory unless otherwise stated. (Vx denotes volume of organ receiving xGy, PRV – planning organ at risk volume, IVC – inferior vena cava)

**5 fractions**		
**Organ**	**Constraint**	**Volume**
Unified viscera constraint (Stomach Duodenum Small bowel Large bowel)	V36	0.1 cc
	V33	0.5 cc
	V25	20cc
Common bile duct	V50	0.5 cc
Liver	Mean	13 Gy
Kidney	Mean to either	10 Gy
Spinal cord PRV	V25<br>V14.5	£0.5 cc<br>£1 cc
Aorta/IVC	V53	0.5 cc
**3 fractions**		
Stomach<br>Duodenum<br>Small bowel<br>Large bowel	V24<br>V22.2	0.5 cc (mandatory)<br>0.5 cc (optimal)
	V20<br>V16.5	5 cc (mandatory)<br>5 cc (optimal)
Common bile duct	V50	0.5 cc
Liver	V15	700 cc
Oesophagus	V25	0.5 cc
Kidneys	V16	200 cc
Spinal cord PRV	V18	0.1 cc
Aorta/IVC	V45	0.5 cc
**1 fraction**		
Duodenum<br>Stomach<br>Small bowel<br>Large bowel	V12<br>V15	0.5 cc (optimal)<br>0.5 cc (mandatory)
	V11	5 cc
Common bile duct	V27	0.5cc
Liver	V9	700 cc
Oesophagus	V19	0.5 cc
Kidneys	V16	200 cc
Spinal cord PRV	V14	0.1 cc
Aorta/IVC	V37	0.5 cc

For peer review only

Treatment delivery

System integrated image registration between the simulation image dataset and the fraction image dataset will be performed. Original plan contours are propagated onto the respective fraction image dataset. All critical structures within a 3 cm axial and 2 cm craniocaudal distance from the surface of the original PTV will be re-contoured on the fraction image dataset. Tumour volume to be re-contoured at clinician discretion.

An estimated delivered dose will be calculated using the software on the console (dose prediction). Subsequently, an adapted radiotherapy plan is generated. During radiation dose delivery, continuous cine MR image acquisition in at least one principal plane (suggested sagittal, but at the discretion of the treating physician) is mandatory for soft tissue tracking and radiation beam gating. To this end, a

tracking slice will be positioned to include a cross-sectional cut of the target or suitable surrogate for intra- fractional soft tissue tracking. The tracking/gating volume will be delineated based on either the GTV or the PTV.

Breathing motion management will be employed. This will include shallow breathing and breath hold. Breath hold may be patient directed or based on staff coaching. Breath-hold assistance devices such as use of mirrors to visualize a wall mounted monitor, MR compatible goggles or image projection into the bore for target positional visualization are allowable and encouraged for use.

In patients receiving single-fraction, the fraction will be sub-divided into 2 pseudo-fractions to allow for a second IGRT match to be carried out. If this match is found to be acceptable and the patient is tolerating the treatment, the rest of the fraction will be delivered immediately. Otherwise, a second adaptive plan could be generated.

Pre-radiotherapy checks

Prospective contour and plan review will be done with an upper-GI radiologist and a second upper-GI site specialist. During treatment, attempts will be made to ensure a second MRL clinician is present. On treatment, secondary quality assurance (QA) contour check will be performed by a trained dosimetrist/medical physicist.

All complex plans must undergo patient specific pre-treatment or on-treatment QA according to local protocols with a fluence and point dose delivery check on the linac recommended using a suitable QA detector array device (e.g. ArcCheck). Pre-treatment QA measurements must use the baseline treatment plan. On-treatment QA should use the most recently delivered treatment fraction.

Analysis plan

A maximum of 60 evaluable patients will be included. There is no formal power calculation as this is a phase I trial, but simulations have been carried out to ensure the sample size is adequate. Conjugate posterior beta distributions will be used to evaluate the safety of five-, three- and single fraction using established dose constraints. The three fractionation regimens will be considered as three separate cohorts and analysed independently. The acceptable toxicity level is defined as 15% for each regimen. If the posterior probability of the DLT rate being above 15% is too high, (e.g. P(risk of DLT > 0.15 | Regimen, Data) > δ with δ defined using simulations before the start of the trial), that regimen will stop for safety. There is no early stopping for success.

A prior beta distribution will be specified for each regimen's toxicity rate. As binary (0=No DLT, 1=DLT) toxicity data is accrued in each regimen, these priors will be updated to conjugate posterior beta distributions. These represent the distribution for the probability of DLT rate within a specific regimen based on all available data. It is from these posterior distributions that inferences about safety will be made. The priors are calibrated to ensure the models provide sensible posterior probabilities based on prior clinical knowledge and incoming trial data.

Only patients for whom we have full information (e.g., experienced a DLT or completed DLT follow-up window) will be included in the modelling. Due to the low acceptable toxicity rate (15%) there is a concern a small number of DLTs early in the trial could result in the trial stopping for safety when in reality the treatment is safe. To reduce the possibility of erroneously stopping early, the model for each regimen will not run until 3 patients from that regimen have full toxicity information. If there are safety concerns prior to this, the trial management group may convene and decide the appropriate action.

Patient and Public Involvement

The Emerald trial has been reviewed and endorsed by patient and carer representatives. Patient and public involvement (PPI) began at the protocol design and development stage. This included a review of the trial patient information sheet and consent form. Further consumer feedback was obtained at the CTRad proposal guidance meeting. Their feedback was adopted and incorporated into the final version of the patient information sheet. PPI representatives will also be invited to attend trial management group meetings throughout the trial.

Author contributions

For peer review only

All contributors meet at least one of the criteria recommended by the ICMJE. ST, TM, and SM conceived the study design. ST, AO, BG, RO, KYC, JD, MR, MP, LS, LG, KN, JG, TM and SM were involved in protocol development and manuscript.

Acknowledgments: The trial is coordinated by the Oncology Clinical Trials Office (OCTO) at the University of Oxford with statistical support from the Centre for Statistics in Medicine, which together form part of the Oxford Clinical Trials Research Unit (OCTRU), a UKCRC Registered Clinical Trials Unit.

Funding: This work is funded through the Oxford University-Genesis Care Collaboration Fund (award/grant number: n/a), the John Black Charitable Foundation (award/grant number: n/a) and Oxford Institute for Radiation Oncology, University of Oxford (award/grant number: n/a). ST is Clinical Career Development Fellow funded by CRUK (award:ART-Net grant). SM is part-funded by Biomedical Research Oxford (award/grant number: n/a). Standard treatment costs for radiotherapy is funded by the NHS (award/grant number: n/a).

Sponsor: The trial is sponsored by the University of Oxford.

Trial registration: ISRCTN10557832

Competing Interest: We declare none of the authors have competing interest.

For peer review only

References

1. Iacobuzio-Donahue CA, Fu B, Yachida S, et al. DPC4 gene status of the primary carcinoma correlates with patterns of failure in patients with pancreatic cancer. *J Clin Oncol* 2009;27(11):1806-13. doi: 10.1200/JCO.2008.17.7188 [published Online First: 20090309]
2. Chuong MD, Springett GM, Freilich JM, et al. Stereotactic body radiation therapy for locally advanced and borderline resectable pancreatic cancer is effective and well tolerated. *Int J Radiat Oncol Biol Phys* 2013;86(3):516-22. doi: 10.1016/j.ijrobp.2013.02.022 [published Online First: 20130405]
3. Mahadevan A, Miksad R, Goldstein M, et al. Induction gemcitabine and stereotactic body radiotherapy for locally advanced nonmetastatic pancreas cancer. *Int J Radiat Oncol Biol Phys* 2011;81(4):e615-22. doi: 10.1016/j.ijrobp.2011.04.045 [published Online First: 20110612]
4. Chang DT, Schellenberg D, Shen J, et al. Stereotactic radiotherapy for unresectable adenocarcinoma of the pancreas. *Cancer* 2009;115(3):665-72. doi: 10.1002/cncr.24059
5. Didolkar MS, Coleman CW, Brenner MJ, et al. Image-guided stereotactic radiosurgery for locally advanced pancreatic adenocarcinoma results of first 85 patients. *J Gastrointest Surg* 2010;14(10):1547-59. doi: 10.1007/s11605-010-1323-7 [published Online First: 20100914]
6. Liauw SL, Ni L, Wu T, et al. A prospective trial of stereotactic body radiation therapy for unresectable pancreatic cancer testing ablative doses. *J Gastrointest Oncol* 2020;11(6):1399-407. doi: 10.21037/jgo-20-187 [published Online First: 2021/01/19]
7. Koong AC, Le QT, Ho A, et al. Phase I study of stereotactic radiosurgery in patients with locally advanced pancreatic cancer. *Int J Radiat Oncol Biol Phys* 2004;58(4):1017-21. doi: 10.1016/j.ijrobp.2003.11.004
8. Schellenberg D, Goodman KA, Lee F, et al. Gemcitabine chemotherapy and single-fraction stereotactic body radiotherapy for locally advanced pancreatic cancer. *Int J Radiat Oncol Biol Phys* 2008;72(3):678-86. doi: 10.1016/j.ijrobp.2008.01.051 [published Online First: 20080418]
9. Hammel P, Huguet F, van Laethem JL, et al. Effect of Chemoradiotherapy vs Chemotherapy on Survival in Patients With Locally Advanced Pancreatic Cancer Controlled After 4 Months of Gemcitabine With or Without Erlotinib: The LAP07 Randomized Clinical Trial. *JAMA* 2016;315(17):1844-53. doi: 10.1001/jama.2016.4324
10. Hurt CN, Falk S, Crosby T, et al. Long-term results and recurrence patterns from SCALOP: a phase II randomised trial of gemcitabine- or capecitabine-based chemoradiation for locally advanced pancreatic cancer. *Br J Cancer* 2017;116(10):1264-70. doi: 10.1038/bjc.2017.95 [published Online First: 20170404]
11. Hoyer M, Roed H, Sengelov L, et al. Phase-II study on stereotactic radiotherapy of locally advanced pancreatic carcinoma. *Radiother Oncol* 2005;76(1):48-53. doi: 10.1016/j.radonc.2004.12.022
12. Schellenberg D, Kim J, Christman-Skieller C, et al. Single-fraction stereotactic body radiation therapy and sequential gemcitabine for the treatment of locally advanced pancreatic cancer. *Int J Radiat Oncol Biol Phys* 2011;81(1):181-8. doi: 10.1016/j.ijrobp.2010.05.006 [published Online First: 20110505]
13. Good J, George B, Teoh S, et al. OC-0112 Feasibility and safety of daily adapted MR-guided SABR for pancreatic cancer in the UK. *Radiotherapy and Oncology* 2022;170:S86-S87. doi: 10.1016/S0167-8140(22)02488-4

14. Rudra S, Jiang N, Rosenberg SA, et al. Using adaptive magnetic resonance image-guided radiation therapy for treatment of inoperable pancreatic cancer. *Cancer Med* 2019;8(5):2123-32. doi: 10.1002/cam4.2100 [published Online First: 20190401]
15. Hall WA, Small C, Paulson E, et al. Magnetic Resonance Guided Radiation Therapy for Pancreatic Adenocarcinoma, Advantages, Challenges, Current Approaches, and Future Directions. *Front Oncol* 2021;11:628155. doi: 10.3389/fonc.2021.628155 [published Online First: 20210511]
16. M. Chuong; K. Mittauer; R. Herrera; T. Romaguera; D. Alvarez; R. Kotecha; M. Hall AKJBJMA. OC-0415 Long-term outcomes of MR-guided SABR & on-table adaptive replanning for unresectable pancreas cancer. *Radiotherapy and Oncology* 2021;161:S310-S11. doi: https://doi.org/10.1016/S0167-8140(21)06902-4.
17. Sivakumar S, Abu-Shah E, Ahern DJ, et al. Activated Regulatory T-Cells, Dysfunctional and Senescent T-Cells Hinder the Immunity in Pancreatic Cancer. *Cancers (Basel)* 2021;13(8) doi: 10.3390/cancers13081776 [published Online First: 20210408]
18. Ye J, Mills BN, Zhao T, et al. Assessing the Magnitude of Immunogenic Cell Death Following Chemotherapy and Irradiation Reveals a New Strategy to Treat Pancreatic Cancer. *Cancer Immunol Res* 2020;8(1):94-107. doi: 10.1158/2326-6066.CIR-19-0373 [published Online First: 20191112]
19. Mills BN, Qiu H, Drage MG, et al. Modulation of the Human Pancreatic Ductal Adenocarcinoma Immune Microenvironment by Stereotactic Body Radiotherapy. *Clin Cancer Res* 2022;28(1):150-62. doi: 10.1158/1078-0432.CCR-21-2495 [published Online First: 20211203]
20. Kluter S. Technical design and concept of a 0.35 T MR-Linac. *Clin Transl Radiat Oncol* 2019;18:98-101. doi: 10.1016/j.ctro.2019.04.007 [published Online First: 20190408]
21. Chu K, Teoh S, Maughan T, et al. PO-1677 Planning feasibility study of three and single fraction Pancreas MR-Linac SBRT - Phase 1 trial setup. *Radiotherapy and Oncology* 2022;170:S1476-S77. doi: 10.1016/S0167-8140(22)03641-6

For peer review only

Figure 1 – Trial recruitment phases. (DLT – dose limiting toxicities).

Figure 2 - Trial schema and overview of follow-up (MDT- multidisciplinary team)

For peer review only

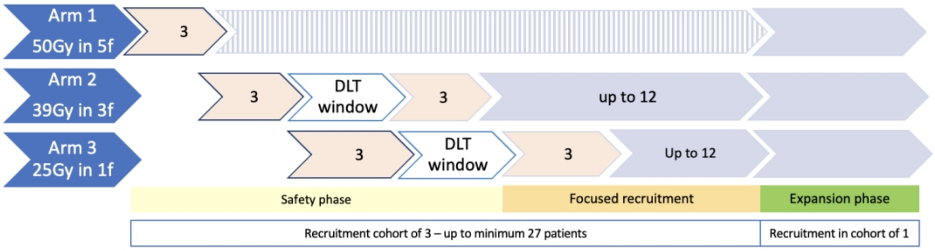

Figure 1 – Trial recruitment phases. (DLT – dose limiting toxicities).

285x83mm (144 x 144 DPI)

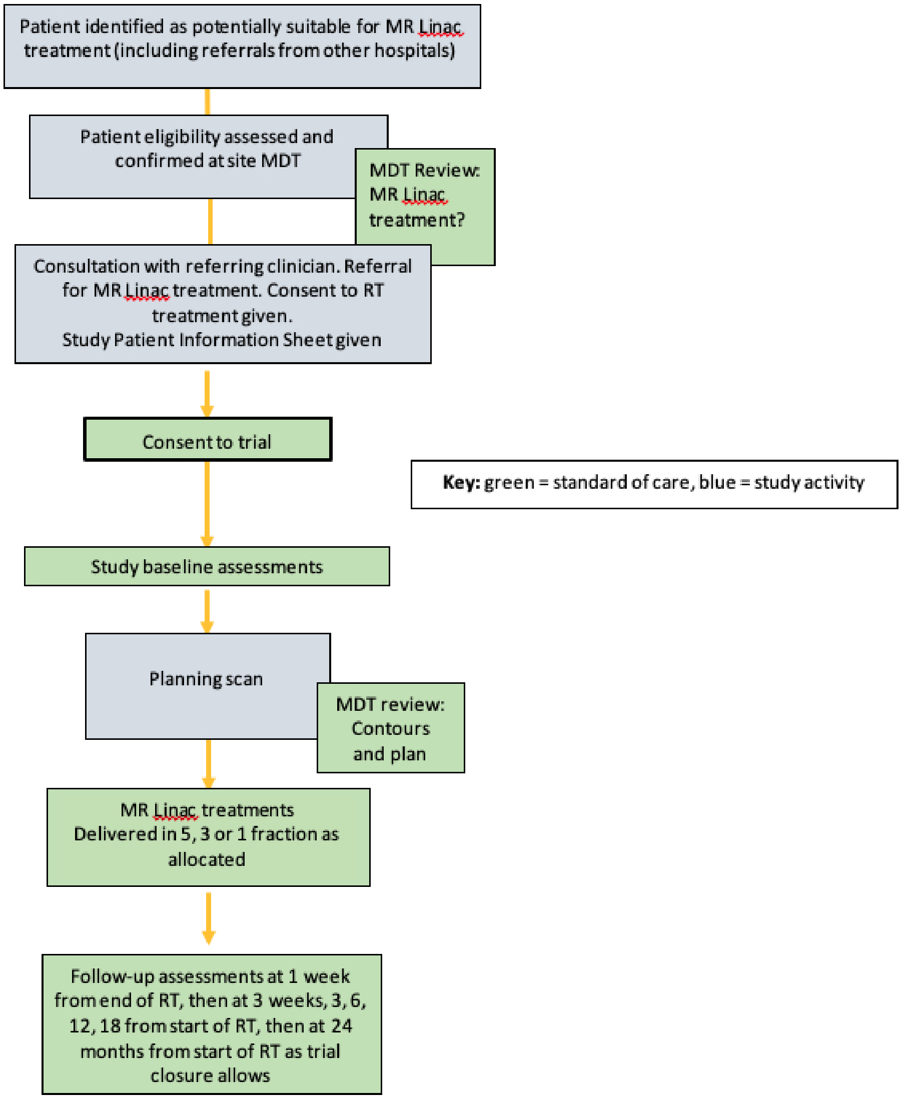

Figure 2 - Trial schema and overview of follow-up (MDT- multidisciplinary team)

213x264mm (72 x 72 DPI)

Eligibility criteria for entry into the main study

Inclusion criteria:

A patient will be eligible for inclusion in this study if all of the following criteria apply.

1. Participants must be fit and scheduled to receive MR guided radiotherapy for pancreatic cancer. There are no specific restrictions on tumour size, number or interval from diagnosis.
2. Localised pancreatic cancer, which may be
   a. locally advanced and inoperable pancreatic cancer
   b. inoperable on medical grounds
   c. operable, but patient declines surgery
   d. locally recurrent pancreatic cancer

3. Histologically proven pancreatic ductal adenocarcinoma or cytological proven pancreatic malignancy. Where histology/cytology is 'suspicious' MDT should confirm that it is appropriate to treat as malignancy.
4. Male or Female, aged 16 years or above.
5. Life expectancy of at least 6 months.
6. ECOG performance status 0- 1.
7. Haematological and biochemical indices within defined ranges:

Lab Test	Value required
Haemoglobin (Hb)	≥ 8.0 g/dL
Platelet count	≥50 x 10^9/l;
Neutrophils	≥ 1.0 x 10^9/l.
Total bilirubin	≤ 1.5 x IULN
AST(SGOT) or ALT(SGPT)	≤ 3.0 x IULN

8. Able (in the investigators' opinion) and willing to comply with all study requirements for the duration of the study.
9. Willing and able to give informed consent.

Exclusion criteria:

A patient will not be eligible for the trial if any of the following apply:

1. Patients with specific MRI exclusion criteria – metallic implants, shrapnel, claustrophobia or other expected intolerance of prolonged (up to 90 minutes) stay in MRI scanner.
2. Prior radiotherapy to the upper abdomen
3. Pregnant or breast-feeding women, or women of childbearing potential unless effective methods of contraception are used. Male patients who do not agree to use a condom during RT treatment and for three months after or who are not surgically sterile.
4. Distant metastatic disease or local disease that cannot be encompassed in the SABR field.

For peer review only

SCHEDULE OF ASSESSMENTS

**Procedure** *(grey shading denotes SoC activities)*	**Pre-screening** Within 3 months of RT #1	**Screening & baseline** Within 28 Days of planning scan	**Planning scan visit**	**RT fraction[14]**					**Post radiotherapy**							**Progression** (if applicable)	**Early Withdrawal** (if applicable)	**End of study[19] (+/-1 months)**
				**#1**	**#2**	**#3**	**#4**	**#5**	**+1 weeks[16] (+/- 4 days)**	**3 weeks[17] (+/- 1 wk)**	**6 weeks[17] (+/- 2 wks)**	**3 months[17] (+2 wks)**	**6 months[17] (+/- 1 mon)**	**12 months[17] (+/- 1 mon)**	**18 months[17] (+/- 1 mon)**			
**CT Scan TAP [1]**	X											X	X	X		X		
**Informed consent [2]**		X																
**Demographics[3]**		X																
**Baseline sign & symptoms[4]**		X																
**Medical history**		X																
**Haematology[5]**		X								X		X						
**Biochemistry[6,7]**		X								X		X						
**Ca19.9 tumour marker**		X								X		X						
**Urine pregnancy test (WOCBP only)**		X																
**Clinical review & disease assessment [8]**		X							X	X	X	X	X	X	X		X	
**Performance status**		X		X[15]					X		X	X	X	X	X			
**AE review [9]**			X[21]	X	X	X	X	X	X	X	X	X	X	X	X		X	X
**DLT review**				X	X	X	X	X	X	X	X	X					X[18]	
**Late-onset severe toxicities review**													X	X	X		X[20]	X
**Planning scan (0.35T MRI)**			X															
**MR Linac treatment [11]**				X	X	X	X	X										
**Optional research blood sample[12]**		X								X		X						
**Data capture: PFS, survival status, resection status, restart of chemotherapy & locoregional failure[13]**												X	X	X [19]	X	X	X [19]	X [19]
**Reason for withdrawal**																	X	

1. Standard of care (SOC) diagnostic CT and optional MRI are used as baseline imaging and whole-body PET-CT if performed in routine care. Further CT scans (& MRI, PET-CT where applicable) at 3, 6 & 12 months follow-up and if relapse/progression occurs are also standard of care. Scans and clinical report to be pseudonymised with trial subject ID and transferred to OU. Scan images and report need to be transferred to the OU if scan carried out at another trust.
2. See section 4.5 for further information on informed consent process.
3. Demographic details to include age and sex
4. Baseline Sign and Symptoms: provide date of onset, event diagnosis (if known) or sign/symptom, severity, time course. Terms should be specific medical terms according to NCI CTCAE version 5. Please avoid using abbreviations, combined terms e.g. nausea and vomiting and ambiguous terms e.g. deranged, abnormal.
5. Haematology: Full Blood Count.
6. eGFR at baseline only.
7. Biochemistry: sodium, potassium, urea and electrolytes, creatinine, ALT or AST, Bilirubin, Albumin, alkaline phosphatase.
8. As considered appropriate by clinician. In follow-up this should include disease assessment for progression
9. AE assessment may be undertaken face to face, by telephone or audio/video call through the internet.
10. The planning scan is required as a routine part of clinical care, for the study an additional set of research images may be acquired during the planning scan.
11. MR Linac treatment will be given in 5, 3 or 1 fractions as per dose selection process detailed in section 10.0 & 10.1.
12. Optional Research Blood Sample: For patients who have consented: Research bloods to be taken pre-treatment, 3 week and 3 months post RT.
13. For participants who have not reached 24 months follow-up at study closure this data will be collected at an earlier timepoint, at least 3 months after start of RT and as close as possible to study closure.
14. Fractions may be missed/delayed at Investigators' discretion
15. ECOG status prior to fraction 1.
16. From the end of radiotherapy
17. From start of radiotherapy
18. If early withdrawal is within 3-month DLT collection window
19. Evaluations to be completed at end of study, 24 months (from RT Fraction #1) follow-up or death
20. If early withdrawal is after 3-month DLT Collection window.
21. The AE Review at the Planning Scan Visit can be performed with the RT Fraction #1 AE review, as long as the review includes AE assessment from the Planning Scan Visit date.

For peer review only

BMJ Open

Evaluation of hypofractionated adaptive radiotherapy using the MR Linac in localised pancreatic cancer: Protocol summary of the Emerald-Pancreas Phase 1/expansion study located at Oxford University Hospital UK.

Journal:	*BMJ Open*
Manuscript ID	bmjopen-2022-068906.R2
Article Type:	Protocol
Date Submitted by the Author:	01-Aug-2023
Complete List of Authors:	Teoh, Suliana; University of Oxford Department of Oncology; Oxford University Hospitals NHS Foundation Trust<br>Ooms, Alexander; University of Oxford, Nuffield Department of Orthopaedics, Rheumatology and Musculoskeletal Disorders<br>George, Ben; GenesisCare UK<br>Owens, Rob; Oxford University Hospitals NHS Foundation Trust; GenesisCare UK<br>Chu, Kwun-Ye; University of Oxford Department of Oncology; Oxford University Hospitals NHS Foundation Trust<br>Drabble, Joe; GenesisCare UK<br>Robinson, Maxwell; Oxford University Hospitals NHS Foundation Trust<br>Parkes, Matthew; Oxford University, Oxford Clinical Trials Research Unit (OCTRU)<br>Swan, Lynda; University of Oxford Department of Oncology<br>Griffiths, Lucinda; University of Oxford Department of Oncology<br>Nugent, Killian; Oxford University Hospitals NHS Foundation Trust, Department of Oncology; GenesisCare UK, Oncology<br>Good, James; GenesisCare UK<br>Maughan, Tim; University of Oxford Department of Oncology; Oxford University Hospitals NHS Foundation Trust<br>Mukherjee, Somnath; University of Oxford Department of Oncology; Oxford University Hospitals NHS Foundation Trust
**Primary Subject Heading**:	Oncology
Secondary Subject Heading:	Gastroenterology and hepatology
Keywords:	Magnetic resonance imaging < RADIOTHERAPY, Hepatobiliary tumours < ONCOLOGY, Radiation oncology < RADIOTHERAPY

SCHOLARONE™
Manuscripts

Title: Evaluation of hypofractionated adaptive radiotherapy using the MR Linac in localised pancreatic cancer: Protocol summary of the Emerald-Pancreas Phase 1/expansion study located at Oxford University Hospital UK.

Authors: S Teoh[1,2], A Ooms[3], B George[4], R Owens[2,3], KY Chu[1,2], J Drabble[4], M Robinson[2], M Parkes[3], L Swan[1], L Griffiths[1], K Nugent[1,4], J Good[4], T Maughan[1], S Mukherjee[1,2]

[1] Department of Oncology, University of Oxford, OX3 7DQ, UK
[2] Oxford University Hospitals NHS Foundation Trust, OX3 7LE, UK
[3] Nuffield Department of Orthopaedics, Rheumatology and Musculoskeletal Disorders, University of Oxford, OX3 7LD, UK
[4] GenesisCare UK, OX4 6LB, UK

Corresponding author: Prof Somnath Mukherjee

Corresponding address:
Department of Oncology
University of Oxford
Old Road Campus Research Building
Roosevelt Drive
Oxford OX3 7DQ

Mobile: +44 7921855863

Email: somnath.mukherjee@oncology.ox.ac.uk

Keywords: Pancreatic cancer, online adaptive radiotherapy, MR-guided radiotherapy, SABR

Word count: 3925

For peer review only

Abstract

Introduction:
Online adaptive magnetic resonance guided radiotherapy (MRgRT) allows for dose escalation to pancreatic cancer while sparing surrounding critical organs. We seek to evaluate the safety of delivering hypofractionated five-, three- and single fraction MR-guided stereotactic ablative radiotherapy (SABR) to the pancreas.

Methods and analysis:
This is a single centre three-arm phase 1 non-randomised safety study. Patients with localised pancreatic cancer will receive either 50Gy in five- (Biological equivalent dose (BED_{10}) =100Gy), 39Gy in three- (BED_{10} =90Gy), or 25Gy in a single fraction (BED_{10} =87.5Gy) MR-guided daily online adaptive radiotherapy. Each fractionation regime will be assessed as independent cohorts to determine tolerability, assessed continuously using Bayesian conjugate posterior beta distributions. The primary endpoint of the study is to establish the safety of five-, three- and single fraction MR-guided hypofractionation SABR in localised pancreatic cancer by assessing dose-limiting toxicities. Secondary endpoints include overall survival, progression free survival, local control rates, overall control rate, resection rates, long-term toxicities and freedom from second line chemotherapy. This study plans to also explore imaging and immune biomarkers that may be useful to predict outcome and personalise treatment. The trial will recruit up to 60 patients with a safety run-in.

Ethics and dissemination: The trial is approved by the West Midlands - Black Country Research Ethics Committee 22/WM/0122.The results will be disseminated via conference presentations, peer-reviewed scientific journals, and submission to regulatory authorities. The data collected for the study, including individual participant data, will be made available to researchers on request to the study team and with appropriate reason, via octo-enquiries@oncology.ox.ac.uk. The shared data will be de-identified participant data and will be available for 3 years following publication of the study. Data will be shared with investigator support, after approval of a proposal and with a signed data access agreement.

Trial registration number: ISRCTN10557832

Registration date: 16/08/2022

Keywords:
Pancreatic cancer, online adaptive radiotherapy, MR-guided radiotherapy, SABR

Strength and Limitations

- This is a phase I safety study evaluating online adaptive extreme hypofractionated radiotherapy using novel MR-guided-radiotherapy (strength).
- There is continuous monitoring of the primary outcome of dose limiting toxicity using conjugate posterior beta distribution (strength).
- Longer term outcome data is limited due to follow-up period of up to 24 months (limitation).
- The trial has a non-comparative design (limitation).
- All three fractionation regimes will be considered as three separate cohorts and therefore outcome could be that three different SABR prescriptions are safe to deliver in pancreatic cancer (strength).

For peer review only - http://bmjopen.bmj.com/site/about/guidelines.xhtml

Background

The role of radiotherapy in patients with inoperable pancreatic cancer is controversial due to its high propensity to metastasize. Meaningful benefit of local control is challenging to quantify when most patients die of distant metastatic disease. Nevertheless, not all patients with adenocarcinoma of the pancreas develop distant metastatic disease. A third of these patients die mainly of local progression [1]. As systemic treatment improves, local control is likely to be increasingly important. Stereotactic ablative radiotherapy (SABR) where an ablative dose of RT is delivered to a small volume in one to five fractions, has been shown to achieve local control rates as high as 80-100% [2-8], compared to about 50-70% reported in CRT trials [9,10]. Initial SABR experiences for treatment of locally advanced pancreatic cancer with ablative doses of radiation had reported high rates of toxicity, particularly with regimens delivered in less than 5 fractions [5,6,11].

There are a number of challenges in delivering highly conformal radiotherapy in pancreatic cancer. Firstly, tumour and organs-at-risk (OAR) visualisation during treatment setup is limited with CT-based imaging. Moreover, the proximity of pancreatic tumours to highly mobile radiosensitive organs such as the duodenum, stomach and bowel means delivering high doses while sparing these organs is difficult. A number of studies have explored the use of SABR to improve outcome. Of concern is the toxicity rate observed in some of these studies. Hoyer et al treated patients with 45Gy in 3 fractions and reported 5 out of 22 patients developing severe mucositis or perforation [11]. Not all patients had on-board CT-imaging for radiotherapy setup. Similarly, Liauw et al reported up to 27% grade 3 and above late toxicities following three-fraction SABR [6]. In both cases large PTV margin and no motion management was utilised. Schellenberg et al evaluated concurrent gemcitabine with single fraction 25 Gy SABR for LAPC and reported freedom from local progression was 88% at 1 year. However, they reported 15% grade 2 ulcers and one patient with a duodenal perforation [12].

Daily online adaptive magnetic resonance imaging (MRI)-guided radiotherapy (MRgRT) is a novel method of delivering radiotherapy that could potentially allow for dose escalation without exceeding dose to the organs-at-risk (OAR). MRI provides superior visualisation of organs such as the stomach, duodenum, and bowel as well as improved tumour identification. Online adaptive MRgRT gives the opportunity to adapt treatment plans daily to account for inter-fraction variability of these organs to maintain target coverage while sparing high doses to OAR [13]. Furthermore, the ability to track the tumour during treatment delivery means ‘real-time’ intra-fraction monitoring is possible.

Early data from a retrospective study shows that dose escalation to 50Gy in 5 fractions using online adaptive MRgRT is safe and may result in improved survival [14]. This retrospective analysis of 42 locally advanced pancreatic cancer patients treated by MR-guided adaptive RT at four institutions demonstrated that high-dose SABR or hypofractionated radiation therapy delivered using daily adaptive dose planning has the potential to further improve overall survival [14]. A control group of 19 patients treated to more conventional radiation doses without frequent dose adaptation showed a median survival of 14.8 months, while patients treated to high radiation doses (n=23, maximum BED10 of >90 Gy) under daily or almost daily adaptive re-planning had an estimated median survival of 27.8 months (p=0.005). Increased radiation dose delivery using daily dose adaptation was correlated with less grade 3 toxicity (0% in the high dose group vs 15.8% in patients treated to lower radiation doses without dose adaptation). Hall et al, summarised the current published experience for MRgRT in pancreatic cancer and found local control rates of between 77-88% at 1 year with toxicity rates of less than 10% (n=141 from 6 studies mostly retrospective) [15]. Chuong et al, retrospectively analysed 50 pancreatic cancer patients treated with 50Gy in 5 fraction using online daily adaptation on the ViewRay MRlinac and found one-year, and 2-year estimated local control were 97.8%, and 88.9%, respectively. Median survival was 21 months (1 and 2-year OS 87.9% and 50%) (ESTRO 2021) [16].

Pancreatic adenocarcinoma features both local and systemic immunosuppression [17]. This enables immune escape and establishment of the tumour both at the primary and distant sites. Whether or not ablative radiation doses to the primary tumour can induce systemic tumour immunity in pancreatic cancer is unknown. In murine pancreatic ductal adenocarcinoma (PDAC) models, SABR has been shown to induce immunogenic cell death (ICD) and promote tumour cell antigen presentation resulting in activation of tumour-specific T cells [18]. Mills et al evaluated the immune response from

For peer review only - http://bmjopen.bmj.com/site/about/guidelines.xhtml

histopathological samples of patients treated with SABR (25Gy in 5 fractions) pre-operatively vs surgery alone and found that SABR reduced PDAC cell density, induced ICD and increased the proportion of PD-1+ T cells. However, despite these finding, intra-tumour T cell levels remain low and outnumbered by myeloid suppressors [19]. While SABR to an unselected population of LAPC patients is unlikely to significantly impact survival outcomes, there is potential for a multi-modality approach with immunotherapy, particularly when combined with biomarkers suggestive of a locally aggressive rather than metastatic phenotype.

The Emerald-Pancreas trial will evaluate the safety of delivering five-, three and single fractions SABR to the pancreas using online adaptive MRgRT. Shorter fractionation schedules are more convenient for patients and increase access to and cost-effectiveness of the scarce and expensive resource of an MRgRT machines. Being able to achieve high rates of local control of the primary tumour with minimal side effects and limited impact on patient lives would be a great advantage for patients. It will also enable greater access for systemic therapies, including conventional chemotherapy and novel immunotherapies, to develop more effective long term control regimens to combat this highly aggressive disease.

Methods/Design

The aim of the trial is to assess safety of extreme hypofractionation of SABR using MRgRT in pancreatic cancer. The five-, three- and single fraction MRgRT treatments will be assessed as independent cohorts to determine if each one is tolerable. Tolerability will be assessed using conjugate posterior beta distributions.

Patient and Public Involvement

The Emerald trial has been reviewed and endorsed by patient and carer representatives. Patient and public involvement (PPI) began at the protocol design and development stage. This included a review of the trial patient information sheet and consent form. Further consumer feedback was obtained at the CTRad proposal guidance meeting. Their feedback was adopted and incorporated into the final version of the patient information sheet. PPI representatives will also be invited to attend trial management group meetings throughout the trial.

Trial design

This is a three-arm, non-randomised, open, safety study. There are three phases of recruitment to the study: an initial safety run-in, a focussed recruitment phase, and an expansion phase (see Figure 1).

In the initial safety run-in, three patients will be recruited into the five-fraction regimen. The three-fraction regimen will open immediately once three patients have been recruited into five-fraction regimen. Once three patients have been accrued to the three-fraction regimen, the single fraction regimen can open immediately. Recruitment to the one- and three-fractions regimens will pause while waiting for 3 months dose limiting toxicity (DLT) follow-up from the first three patients in each cohort. It is possible both the single and three fraction regimens will be paused at the same time. Any patients recruited whilst both one- and three-fractions regimens are paused will be assigned the five-fraction regimen.

Following the safety run-in, the focussed recruitment will start. Recruitment will continue in cohorts of 3 alternating between the three- and one-fraction regimens until a total of 12 patients in the one- and three-fraction regimens have been recruited. Recruitment to this phase will be continuous. After 3 patients' full DLT follow-up data in any regimen, the beta binomial model for that regimen can be run to assess the safety stopping rule in the event of a DLT.

For the remainder of the trial, the expansion phase recruitment will be in cohorts of 1 to all three regimens and recruitment will be continuous, alternating between one-, three-, and five-fractions. Where plans that meet dose constraints are unable to be generated for single fraction regimens that patient will be assigned to the three-fraction regimen or alternatively the five-fraction regimen if recruitment to the three-fraction cohort is paused or closed. Where plans that meet dose constraints

For peer review only - http://bmjopen.bmj.com/site/about/guidelines.xhtml

are unable to be generated for three-fraction regimens, that patient will be assigned to the five-fraction regimen. These patients will be included in the main analysis for the regimen they are treated in. A sensitivity analysis will be performed excluding these patients as they may be at higher risk of toxicity. Figure 2 shows the trial schema and overview of follow-up. The duration of the study will be up to 24 months from patient starting radiotherapy. All participants will be followed up for at least 3 months and to the maximum time until study closure. The trial has been open to recruitment since August 2022 with the first patient first visit (FPFV) being on 25th of August 2022 with the end of study date being the 31st of December 2024. Our last patient first visit (LPFV) is planned for the 30th of June 2024.

Trial intervention

Patients will receive radiotherapy online adaptive MRgRT on Viewray MRIdian MR Linac [20]. True Fast Imaging with Steady State Precession (TRUFISP) T2/T1weightedsequences are used to provide the cine MRI (real-time imaging) for beam gating, and volumetric imaging for OARs. SABR fractions will be delivered ideally on alternate days, treating at least twice per week. Daily treatment is permissible with at least 18 hours between fractions for five-fraction regimen.

Hypofractionation

For each of the three fractionations (50Gy in five fractions, 39Gy in three fractions or 25Gy in a single fraction) we have defined a single dose level (See Table 1). The starting regimen will be 50 Gy in 5 fractions (BED10 =100 Gy). Our in-silico planning study showed that three and single-fraction pancreas SABR plans could be generated while meeting organ dose constraints and delivering a meaningful dose to the target. Furthermore, treatments could be delivered within a reasonable timeframe [21]. Patients included in the study were from the CAP cohort (n=8, median GTV 41.35cc, (range 15.9-64.4). The median PTV V100 coverage for 39Gy in three fractions and 25Gy in one fraction was 75.7% (60.6-91.6%) and 66.1 (60.1-84.2%) respectively. The median treatment delivery times for 15.2min (12.5-21.7min) and 21.0min (15.9-33.2min) for 39Gy in three fractions and 25Gy in a single fraction respectively.

Table 1 – Table 1: Radiotherapy dose levels. BED 10 biological equivalent dose for acute reacting tissues (α/β=10).

Fractions	Dose to PTV (Gy)		
	Dose/#	Total dose	BED 10
5	10	50	100
3	13	39	90
1	25	25	88

Dose limiting toxicity (DLT)

In this study, DLT is defined in the following list of possible SABR treatment related adverse events (defined according to CTCAE v5.0) seen in the period from starting SABR treatment to three months post treatment.

DLT Events (within 3 months)

- Grade 3 upper gastro-intestinal (GI) bleeding
- GI fistula (any grade)
- Grade 4 nausea/vomiting uncontrolled despite optimum anti-emetics
- Grade 4 pancreatitis not stent related
- Vascular events (where these are not considered to be tumour related)

For peer review only - http://bmjopen.bmj.com/site/about/guidelines.xhtml

Late-onset severe toxicities
Late-onset severe toxicities may occur and will be monitored for during follow up (>3 months and up to 24 months where trial remains open and/or patient on trial)

- >=Grade 3 upper GI bleed
- gastro-intestinal fistula (any grade)
- >= Grade 3 vascular events

Trial population
The target population for the Emerald-Pancreas trial consists of male or female aged 16 years or above with an ECOG performance status of 0-1 with a localised pancreatic cancer. Patients are not permitted to enter the trial if they have a contraindication to having an MRI (presence of metallic implants, shrapnel, claustrophobia, or other expected intolerance of prolonged stay in an MRI scanner). The trial is based at a single UK centre (Oxford University Hospital NHS Foundation Trust).

There are no specific restrictions on tumour size, number or interval from diagnosis. The following patients with localised pancreatic cancer may enter the trial: locally advanced and inoperable pancreatic cancer, inoperable on medical grounds, operable, but declines surgery and locally recurrent pancreatic cancer.

A full list of inclusion and exclusion criteria can be found in Appendix 1. At least 3 months of chemotherapy prior to RT is recommended but not mandated. Chemotherapy should be avoided for at least 2 weeks before and for 4 weeks after RT.

Study objectives and endpoints
The primary objective of the study is to establish the safety of five-, three- and single fraction MR-guided hypofractionation SABR in localised pancreatic cancer by assessing DLTs up to three months from start of radiotherapy. The secondary objectives include assessment of efficacy and long-term toxicity with the following endpoints: overall survival, progression free survival, local control rates, overall control rate, resection rates, long-term toxicities, and freedom from second line chemotherapy.

Planned Exploratory objectives for Emerald-Pancreas trial will include evaluation of imaging and immune biomarkers that may be useful to predict outcome from radiotherapy. Research blood samples will be collected baseline, 3 weeks and 3 months after the first RT fraction to monitor changes in immune cell markers in response to treatment in a cohort of up to 20 patients. The schedule for collection of data for the purposes of evaluating all trial endpoints is found in Appendix 2.

Radiotherapy planning
Patients will undergo a dedicated MR-Linac simulation as well as a CT simulation. CT simulation is to be used as primary data set for electron density information for planning dose calculation purposes. Alternatively, creation of synthetic CT simulation is acceptable where a suitable review of dosimetric accuracy using this approach has been carried out. Patient will be fasted four hours prior to scan and treatment. Intravenous contrast is required for the planning CT but not the planning MRI. Patient will be scanned in breath hold. A suitable tracking structure will be identified, either tumour in the first instance or suitable surrogate where tumour tracking is determined unsuitable by clinical team. Ability to track motion will be assessed through MR cine imaging.

The planning CT scan should ideally be carried out post MR simulation. Scan will be in the same breath hold used during MR simulation to maximise the chance of accurate deformation of the CT for use in planning calculations.

Delineation
Tumour volume definition is discussed and peer-reviewed with a pancreatic/upper GI radiologist and a second upper GI clinical oncologist. Outlining of the volumes of interest should take account of all available diagnostic imaging. Gross tumour volume (GTV) should not be reduced for a region that is negative on PET-CT but identified as abnormal on other imaging modalities. The following structures are to be created:

For peer review only - http://bmjopen.bmj.com/site/about/guidelines.xhtml

GTV_T = Macroscopic pancreatic tumour visible on imaging.

GTV_N = peritumoural lymph nodes >1 cm in short axis diameter and likely involved on diagnostic imaging. Include nodes < 1cm if considered involved by radiologist. Prophylactic nodal irradiation is not typically performed.

GTV_Final = GTV_T + GTV_N.

Clinical target volume (CTV) = GTV + 2 mm, with CTV cropped to the visceral OARs.

Planning target volume (PTV) = CTV + 3 mm.

The PTV will be divided into PTV_high and PTV_low so that any overlap of OAR and PTV still allows tolerance of OAR to be achieved. The overlap region needs to be designed and setup as rule so that it can be updated during plan adaptation. Nomenclature is as follows:

Critical structures (CS) = duodenum + small bowel + large bowel + stomach

PTV_high = PTV – (CS + 5 mm*)

PTV_low = intersection between PTV and (CS +5 mm*)

*A 5 - 8 mm margin may be needed from critical structures to achieve plan objectives.

Organs at risk defined below are to be contoured, plus any others considered relevant for the particular case as deemed by CI:

- Duodenum, stomach, small bowel, large bowel and oesophagus:
  Should be outlined on all slices from 3cm above to 3cm below the PTV. Individual loops of duodenum, small bowel and large bowel should be outlined.
- Kidneys
- Liver
- Spinal cord:
- Common bile duct, vessels and gallbladder are to be contoured when near to or overlapping the PTV to avoid hotspots.

Radiotherapy planning
Coplanar step-and-shoot IMRT plans are generated in a pseudo-arc formation. The planning objective is that 98% of PTV_high receives ≥95% of the prescribed dose. If the mandatory duodenum, bowel or stomach constraints cannot be met, PTV coverage should be reduced until the constraints are met. PTV_high coverage should not ideally need compromising, but coverage should also be reduced until mandatory constraints are met where needed. The minimum dose coverage objective of PTV V100%≥60%. The maximum dose in 1 cc of the PTV will be ≥125% and ≤ 140%. Hotspots should be within the GTV. OAR constraints are found in Table 2. All constraints are mandatory unless otherwise stated.

For peer review only - http://bmjopen.bmj.com/site/about/guidelines.xhtml

Table 2: Organs-at-risk dose constraints. All constraints are mandatory unless otherwise stated. (Vx denotes volume of organ receiving xGy, PRV – planning organ at risk volume, IVC – inferior vena cava)

**5 fractions**		
**Organ**	**Constraint**	**Volume**
Unified viscera constraint (Stomach Duodenum Small bowel Large bowel)	V36	0.1 cc
	V33	0.5 cc
	V25	20cc
Common bile duct	V50	0.5 cc
Liver	Mean	13 Gy
Kidney	Mean to either	10 Gy
Spinal cord PRV	V25<br>V14.5	0.5 cc<br>1 cc
Aorta/IVC	V53	0.5 cc
**3 fractions**		
Stomach Duodenum Small bowel Large bowel	V24<br>V22.2	0.5 cc (mandatory)<br>0.5 cc (optimal)
	V20<br>V16.5	5 cc (mandatory)<br>5 cc (optimal)
Common bile duct	V50	0.5 cc
Liver	V15	700 cc
Oesophagus	V25	0.5 cc
Kidneys	V16	200 cc
Spinal cord PRV	V18	0.1 cc
Aorta/IVC	V45	0.5 cc
**1 fraction**		
Duodenum Stomach Small bowel Large bowel	V12<br>V15	0.5 cc (optimal)<br>0.5 cc (mandatory)
	V11	5 cc
Common bile duct	V27	0.5cc
Liver	V9	700 cc
Oesophagus	V19	0.5 cc
Kidneys	V16	200 cc
Spinal cord PRV	V14	0.1 cc
Aorta/IVC	V37	0.5 cc

Treatment delivery

System integrated image registration between the simulation image dataset and the fraction image dataset will be performed. Original plan contours are propagated onto the respective fraction image dataset. All critical structures within a 3 cm axial and 2 cm craniocaudal distance from the surface of the original PTV will be re-contoured on the fraction image dataset. Tumour volume to be re-contoured at clinician discretion.

An estimated delivered dose will be calculated using the software on the console (dose prediction). Subsequently, an adapted radiotherapy plan is generated. During radiation dose delivery, continuous cine MR image acquisition in at least one principal plane (suggested sagittal, but at the discretion of the treating physician) is mandatory for soft tissue tracking and radiation beam gating. To this end, a tracking slice will be positioned to include a cross-sectional cut of the target or suitable surrogate for intra- fractional soft tissue tracking. The tracking/gating volume will be delineated based on either the

For peer review only

For peer review only - http://bmjopen.bmj.com/site/about/guidelines.xhtml

GTV or the PTV.

Breathing motion management will be employed. This will include shallow breathing and breath hold. Breath hold may be patient directed or based on staff coaching. Breath-hold assistance devices such as use of mirrors to visualize a wall mounted monitor, MR compatible goggles or image projection into the bore for target positional visualization are allowable and encouraged for use.

In patients receiving single-fraction, the fraction will be sub-divided into 2 pseudo-fractions to allow for a second IGRT match to be carried out. If this match is found to be acceptable and the patient is tolerating the treatment, the rest of the fraction will be delivered immediately. Otherwise, a second adaptive plan could be generated.

Pre-radiotherapy checks

Prospective contour and plan review will be done with an upper-GI radiologist and a second upper-GI site specialist. During treatment, attempts will be made to ensure a second MRL clinician is present. On treatment, secondary quality assurance (QA) contour check will be performed by a trained dosimetrist/medical physicist.

All complex plans must undergo patient specific pre-treatment or on-treatment QA according to local protocols with a fluence and point dose delivery check on the linac recommended using a suitable QA detector array device (e.g. ArcCheck). Pre-treatment QA measurements must use the baseline treatment plan. On-treatment QA should use the most recently delivered treatment fraction.

Analysis plan

A maximum of 60 evaluable patients will be included. There is no formal power calculation as this is a phase I trial, but simulations have been carried out to ensure the sample size is adequate. Conjugate posterior beta distributions will be used to evaluate the safety of five-, three- and single fraction using established dose constraints. The three fractionation regimens will be considered as three separate cohorts and analysed independently. The acceptable toxicity level is defined as 15% for each regimen. If the posterior probability of the DLT rate being above 15% is too high, (e.g. $P(\text{risk of DLT} > 0.15 \mid \text{Regimen, Data}) > \delta$ with δ defined using simulations before the start of the trial), that regimen will stop for safety. There is no early stopping for success.

A prior beta distribution will be specified for each regimen's toxicity rate. As binary (0=No DLT, 1=DLT) toxicity data is accrued in each regimen, these priors will be updated to conjugate posterior beta distributions. These represent the distribution for the probability of DLT rate within a specific regimen based on all available data. It is from these posterior distributions that inferences about safety will be made. The priors are calibrated to ensure the models provide sensible posterior probabilities based on prior clinical knowledge and incoming trial data.

Only patients for whom we have full information (e.g., experienced a DLT or completed DLT follow-up window) will be included in the modelling. Due to the low acceptable toxicity rate (15%) there is a concern a small number of DLTs early in the trial could result in the trial stopping for safety when in reality the treatment is safe. To reduce the possibility of erroneously stopping early, the model for each regimen will not run until 3 patients from that regimen have full toxicity information. If there are safety concerns prior to this, the trial management group may convene and decide the appropriate action.

Ethics and Dissemination: The trial is approved by the West Midlands - Black Country Research Ethics Committee 22/WM/0122.The results will be disseminated via conference presentations, peer-reviewed scientific journals, and submission to regulatory authorities. The data collected for the study, including individual participant data, will be made available to researchers on request to the study team and with appropriate reason, via octo-enquiries@oncology.ox.ac.uk. The shared data will be de-identified participant data and will be available for 3 years following publication of the study. Data will be shared with investigator support, after approval of a proposal and with a signed data access agreement.

For peer review only - http://bmjopen.bmj.com/site/about/guidelines.xhtml

Author contributions

All contributors meet at least one of the criteria recommended by the ICMJE. ST, TM, and SM conceived the study design. ST, AO, BG, RO, KYC, JD, MR, MP, LS, LG, KN, JG, TM and SM were involved in protocol development and manuscript.

Acknowledgments: The trial is coordinated by the Oncology Clinical Trials Office (OCTO) at the University of Oxford with statistical support from the Centre for Statistics in Medicine, which together form part of the Oxford Clinical Trials Research Unit (OCTRU), a UKCRC Registered Clinical Trials Unit.

Funding: This work is funded through the Oxford University-Genesis Care Collaboration Fund (award/grant number: n/a), the John Black Charitable Foundation (award/grant number: n/a) and Oxford Institute for Radiation Oncology, University of Oxford (award/grant number: n/a). ST is Clinical Career Development Fellow funded by CRUK (award:ART-Net grant). SM is part-funded by Biomedical Research Oxford (award/grant number: n/a). Standard treatment costs for radiotherapy are funded by the NHS (award/grant number: n/a).

Sponsor: The trial is sponsored by the University of Oxford. E-mail: ctrg@admin.ox.ac.uk

Trial registration: ISRCTN10557832

Competing Interest: We declare none of the authors have competing interest.

For peer review only

References

1. Iacobuzio-Donahue CA, Fu B, Yachida S, et al. DPC4 gene status of the primary carcinoma correlates with patterns of failure in patients with pancreatic cancer. *J Clin Oncol* 2009;27(11):1806-13. doi: 10.1200/JCO.2008.17.7188 [published Online First: 20090309]
2. Chuong MD, Springett GM, Freilich JM, et al. Stereotactic body radiation therapy for locally advanced and borderline resectable pancreatic cancer is effective and well tolerated. *Int J Radiat Oncol Biol Phys* 2013;86(3):516-22. doi: 10.1016/j.ijrobp.2013.02.022 [published Online First: 20130405]
3. Mahadevan A, Miksad R, Goldstein M, et al. Induction gemcitabine and stereotactic body radiotherapy for locally advanced nonmetastatic pancreas cancer. *Int J Radiat Oncol Biol Phys* 2011;81(4):e615-22. doi: 10.1016/j.ijrobp.2011.04.045 [published Online First: 20110612]
4. Chang DT, Schellenberg D, Shen J, et al. Stereotactic radiotherapy for unresectable adenocarcinoma of the pancreas. *Cancer* 2009;115(3):665-72. doi: 10.1002/cncr.24059
5. Didolkar MS, Coleman CW, Brenner MJ, et al. Image-guided stereotactic radiosurgery for locally advanced pancreatic adenocarcinoma results of first 85 patients. *J Gastrointest Surg* 2010;14(10):1547-59. doi: 10.1007/s11605-010-1323-7 [published Online First: 20100914]
6. Liauw SL, Ni L, Wu T, et al. A prospective trial of stereotactic body radiation therapy for unresectable pancreatic cancer testing ablative doses. *J Gastrointest Oncol* 2020;11(6):1399-407. doi: 10.21037/jgo-20-187 [published Online First: 2021/01/19]
7. Koong AC, Le QT, Ho A, et al. Phase I study of stereotactic radiosurgery in patients with locally advanced pancreatic cancer. *Int J Radiat Oncol Biol Phys* 2004;58(4):1017-21. doi: 10.1016/j.ijrobp.2003.11.004
8. Schellenberg D, Goodman KA, Lee F, et al. Gemcitabine chemotherapy and single-fraction stereotactic body radiotherapy for locally advanced pancreatic cancer. *Int J Radiat Oncol Biol Phys* 2008;72(3):678-86. doi: 10.1016/j.ijrobp.2008.01.051 [published Online First: 20080418]
9. Hammel P, Huguet F, van Laethem JL, et al. Effect of Chemoradiotherapy vs Chemotherapy on Survival in Patients With Locally Advanced Pancreatic Cancer Controlled After 4 Months of Gemcitabine With or Without Erlotinib: The LAP07 Randomized Clinical Trial. *JAMA* 2016;315(17):1844-53. doi: 10.1001/jama.2016.4324
10. Hurt CN, Falk S, Crosby T, et al. Long-term results and recurrence patterns from SCALOP: a phase II randomised trial of gemcitabine- or capecitabine-based chemoradiation for locally advanced pancreatic cancer. *Br J Cancer* 2017;116(10):1264-70. doi: 10.1038/bjc.2017.95 [published Online First: 20170404]
11. Hoyer M, Roed H, Sengelov L, et al. Phase-II study on stereotactic radiotherapy of locally advanced pancreatic carcinoma. *Radiother Oncol* 2005;76(1):48-53. doi: 10.1016/j.radonc.2004.12.022
12. Schellenberg D, Kim J, Christman-Skieller C, et al. Single-fraction stereotactic body radiation therapy and sequential gemcitabine for the treatment of locally advanced pancreatic cancer. *Int J Radiat Oncol Biol Phys* 2011;81(1):181-8. doi: 10.1016/j.ijrobp.2010.05.006 [published Online First: 20110505]
13. Good J, George B, Teoh S, et al. OC-0112 Feasibility and safety of daily adapted MR-guided SABR for pancreatic cancer in the UK. *Radiotherapy and Oncology* 2022;170:S86-S87. doi: 10.1016/S0167-8140(22)02488-4

For peer review only - http://bmjopen.bmj.com/site/about/guidelines.xhtml

14. Rudra S, Jiang N, Rosenberg SA, et al. Using adaptive magnetic resonance image-guided radiation therapy for treatment of inoperable pancreatic cancer. *Cancer Med* 2019;8(5):2123-32. doi: 10.1002/cam4.2100 [published Online First: 20190401]
15. Hall WA, Small C, Paulson E, et al. Magnetic Resonance Guided Radiation Therapy for Pancreatic Adenocarcinoma, Advantages, Challenges, Current Approaches, and Future Directions. *Front Oncol* 2021;11:628155. doi: 10.3389/fonc.2021.628155 [published Online First: 20210511]
16. M. Chuong; K. Mittauer; R. Herrera; T. Romaguera; D. Alvarez; R. Kotecha; M. Hall AKJBJMA. OC-0415 Long-term outcomes of MR-guided SABR & on-table adaptive replanning for unresectable pancreas cancer. *Radiotherapy and Oncology* 2021;161:S310-S11. doi: https://doi.org/10.1016/S0167-8140(21)06902-4.
17. Sivakumar S, Abu-Shah E, Ahern DJ, et al. Activated Regulatory T-Cells, Dysfunctional and Senescent T-Cells Hinder the Immunity in Pancreatic Cancer. *Cancers (Basel)* 2021;13(8) doi: 10.3390/cancers13081776 [published Online First: 20210408]
18. Ye J, Mills BN, Zhao T, et al. Assessing the Magnitude of Immunogenic Cell Death Following Chemotherapy and Irradiation Reveals a New Strategy to Treat Pancreatic Cancer. *Cancer Immunol Res* 2020;8(1):94-107. doi: 10.1158/2326-6066.CIR-19-0373 [published Online First: 20191112]
19. Mills BN, Qiu H, Drage MG, et al. Modulation of the Human Pancreatic Ductal Adenocarcinoma Immune Microenvironment by Stereotactic Body Radiotherapy. *Clin Cancer Res* 2022;28(1):150-62. doi: 10.1158/1078-0432.CCR-21-2495 [published Online First: 20211203]
20. Kluter S. Technical design and concept of a 0.35 T MR-Linac. *Clin Transl Radiat Oncol* 2019;18:98-101. doi: 10.1016/j.ctro.2019.04.007 [published Online First: 20190408]
21. Chu K, Teoh S, Maughan T, et al. PO-1677 Planning feasibility study of three and single fraction Pancreas MR-Linac SBRT - Phase 1 trial setup. *Radiotherapy and Oncology* 2022;170:S1476-S77. doi: 10.1016/S0167-8140(22)03641-6

For peer review only

Figure 1 – Trial recruitment phases. (DLT – dose limiting toxicities).

Figure 2 - Trial schema and overview of follow-up (MDT- multidisciplinary team)

For peer review only

For peer review only - http://bmjopen.bmj.com/site/about/guidelines.xhtml

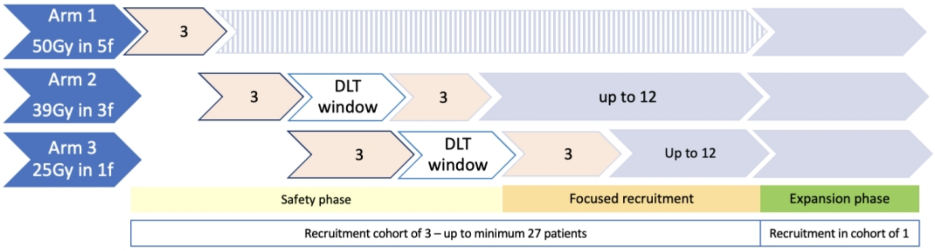

Figure 1 – Trial recruitment phases. (DLT – dose limiting toxicities).

285x83mm (144 x 144 DPI)

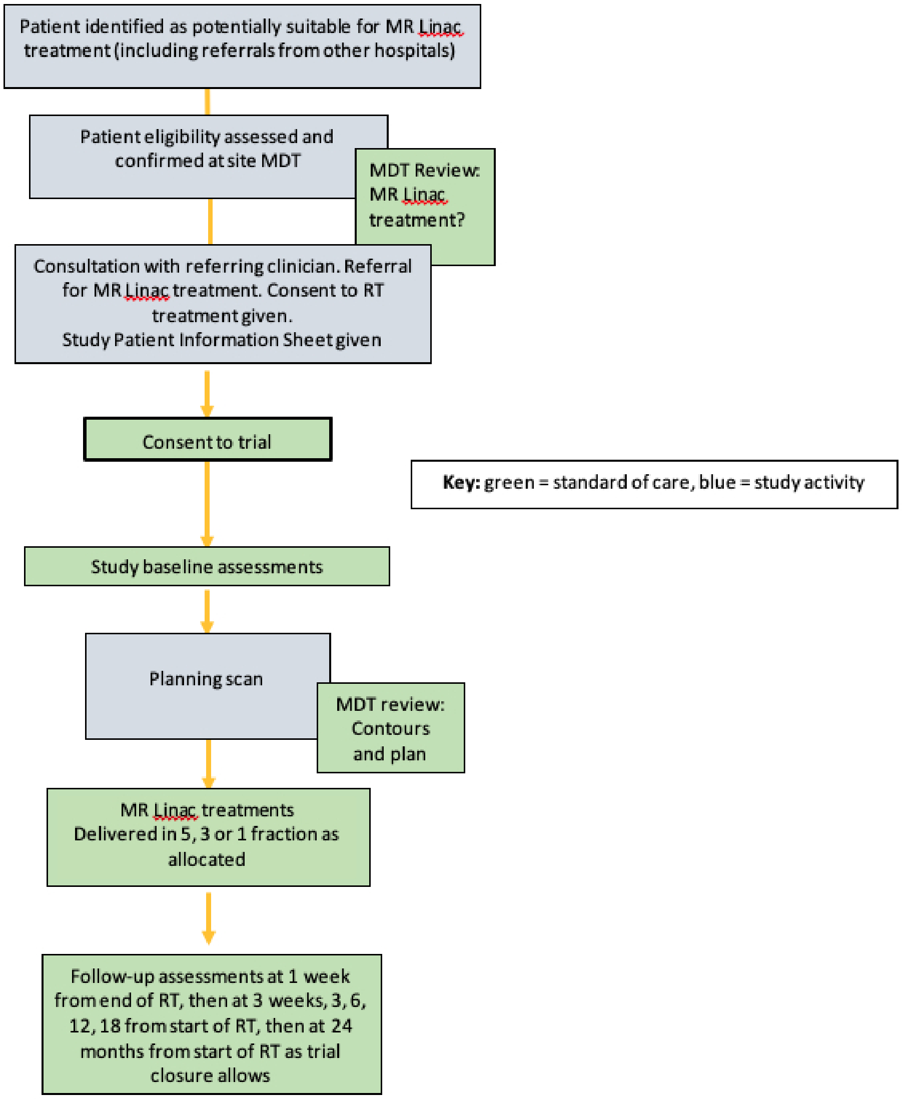

Figure 2 - Trial schema and overview of follow-up (MDT- multidisciplinary team)

213x264mm (72 x 72 DPI)

Eligibility criteria for entry into the main study

Inclusion criteria:

A patient will be eligible for inclusion in this study if all of the following criteria apply.

1. Participants must be fit and scheduled to receive MR guided radiotherapy for pancreatic cancer. There are no specific restrictions on tumour size, number or interval from diagnosis.
2. Localised pancreatic cancer, which may be
   a. locally advanced and inoperable pancreatic cancer
   b. inoperable on medical grounds
   c. operable, but patient declines surgery
   d. locally recurrent pancreatic cancer

3. Histologically proven pancreatic ductal adenocarcinoma or cytological proven pancreatic malignancy. Where histology/cytology is 'suspicious' MDT should confirm that it is appropriate to treat as malignancy.
4. Male or Female, aged 16 years or above.
5. Life expectancy of at least 6 months.
6. ECOG performance status 0- 1.
7. Haematological and biochemical indices within defined ranges:

Lab Test	Value required
Haemoglobin (Hb)	≥ 8.0 g/dL
Platelet count	≥50 x 10^9/l;
Neutrophils	≥ 1.0 x 10^9/l.
Total bilirubin	≤ 1.5 x IULN
AST(SGOT) or ALT(SGPT)	≤ 3.0 x IULN

8. Able (in the investigators' opinion) and willing to comply with all study requirements for the duration of the study.
9. Willing and able to give informed consent.

Exclusion criteria:

A patient will not be eligible for the trial if any of the following apply:

1. Patients with specific MRI exclusion criteria – metallic implants, shrapnel, claustrophobia or other expected intolerance of prolonged (up to 90 minutes) stay in MRI scanner.
2. Prior radiotherapy to the upper abdomen
3. Pregnant or breast-feeding women, or women of childbearing potential unless effective methods of contraception are used. Male patients who do not agree to use a condom during RT treatment and for three months after or who are not surgically sterile.
4. Distant metastatic disease or local disease that cannot be encompassed in the SABR field.

For peer review only

SCHEDULE OF ASSESSMENTS

**Procedure** *(grey shading denotes SoC activities)*	**Pre-screening** Within 3 months of RT #1	**Screening & baseline** Within 28 Days of planning scan	**Planning scan visit**	**RT fraction[14]**					**Post radiotherapy**							**Progress-ion** (if applicable)	**Early Withdra-wal** (if applicable)	**End of study[19]** (+/-1 months)
				**#1**	**#2**	**#3**	**#4**	**#5**	**+1 weeks[16] (+/- 4 days)**	**3 weeks[17] (+/- 1 wk)**	**6 weeks[17] (+/- 2 wks)**	**3 months[17] (+2 wks)**	**6 months[17] (+/- 1 mon)**	**12 months[17] (+/- 1 mon)**	**18 months[17] (+/- 1 mon)**			
**CT Scan TAP [1]**	X											X	X	X		X		
**Informed consent [2]**		X																
**Demographics[3]**		X																
**Baseline sign & symptoms[4]**		X																
**Medical history**		X																
**Haematology[5]**		X								X		X						
**Biochemistry[6,7]**		X								X		X						
**Ca19.9 tumour marker**		X								X		X						
**Urine pregnancy test (WOCBP only)**		X																
**Clinical review & disease assessment [8]**		X							X	X	X	X	X	X	X		X	
**Performance status**		X		X[15]					X	X	X	X	X	X	X			
**AE review [9]**			X[21]	X	X	X	X	X	X	X	X	X	X	X	X		X	X
**DLT review**				X	X	X	X	X	X	X	X	X					X[18]	
**Late-onset severe toxicities review**													X	X	X		X[20]	X
**Planning scan (0.35T MRI)**			X															
**MR Linac treatment [11]**				X	X	X	X	X										
**Optional research blood sample[12]**		X								X		X						
**Data capture: PFS, survival status, resection status, restart of chemotherapy & locoregional failure[13]**												X	X	X [19]	X	X	X [19]	X [19]
**Reason for withdrawal**																	X	

1. Standard of care (SOC) diagnostic CT and optional MRI are used as baseline imaging and whole-body PET-CT if performed in routine care. Further CT scans (& MRI, PET-CT where applicable) at 3, 6 & 12 months follow-up and if relapse/progression occurs are also standard of care. Scans and clinical report to be pseudonymised with trial subject ID and transferred to OU. Scan images and report need to be transferred to the OU if scan carried out at another trust.
2. See section 4.5 for further information on informed consent process.
3. Demographic details to include age and sex
4. Baseline Sign and Symptoms: provide date of onset, event diagnosis (if known) or sign/symptom, severity, time course. Terms should be specific medical terms according to NCI CTCAE version 5. Please avoid using abbreviations, combined terms e.g. nausea and vomiting and ambiguous terms e.g. deranged, abnormal.
5. Haematology: Full Blood Count.
6. eGFR at baseline only.
7. Biochemistry: sodium, potassium, urea and electrolytes, creatinine, ALT or AST, Bilirubin, Albumin, alkaline phosphatase.
8. As considered appropriate by clinician. In follow-up this should include disease assessment for progression
9. AE assessment may be undertaken face to face, by telephone or audio/video call through the internet.
10. The planning scan is required as a routine part of clinical care, for the study an additional set of research images may be acquired during the planning scan.
11. MR Linac treatment will be given in 5, 3 or 1 fractions as per dose selection process detailed in section 10.0 & 10.1.
12. Optional Research Blood Sample: For patients who have consented: Research bloods to be taken pre-treatment, 3 week and 3 months post RT.
13. For participants who have not reached 24 months follow-up at study closure this data will be collected at an earlier timepoint, at least 3 months after start of RT and as close as possible to study closure.
14. Fractions may be missed/delayed at Investigators' discretion
15. ECOG status prior to fraction 1.
16. From the end of radiotherapy
17. From start of radiotherapy
18. If early withdrawal is <u>within</u> 3-month DLT collection window
19. Evaluations to be completed at end of study, 24 months (from RT Fraction #1) follow-up or death
20. If early withdrawal is <u>after</u> 3-month DLT Collection window.
21. The AE Review at the Planning Scan Visit can be performed with the RT Fraction #1 AE review, as long as the review includes AE assessment from the Planning Scan Visit date.

For peer review only

STANDARD PROTOCOL ITEMS: RECOMMENDATIONS FOR INTERVENTIONAL TRIALS

SPIRIT 2013 Checklist: Recommended items to address in a clinical trial protocol and related documents*

Section/item	Item No	Description
**Administrative information**		
Title	1	Descriptive title identifying the study design, population, interventions, and, if applicable, trial acronym<br>Main manuscript Page 1 Line 1<br>Emerald_Protocol_V2.0 28Jun2022 Page 1
Trial registration	2a	Trial identifier and registry name. If not yet registered, name of intended registry<br>Main manuscript Page 2 Line 60<br>Emerald_Protocol_V2.0 28Jun2022 Page 1
	2b	All items from the World Health Organization Trial Registration Data Set<br>ISRCTN registered trial:<br>Main manuscript Page 2 Line 60<br>Main Manuscript Page 2 Line 62<br>Emerald_Protocol_V2.0 28Jun2022 Page 1
Protocol version	3	Date and version identifier<br>Emerald_Protocol_V2.0 28Jun2022
Funding	4	Sources and types of financial, material, and other support<br>Main manuscript Page 10 Line 426 to 431<br>Emerald_Protocol_V2.0 28Jun2022 Page 45
Roles and responsibilities	5a	Names, affiliations, and roles of protocol contributors<br>Main manuscript Page 1 Line 4 to 12<br>Main manuscript Pages 9 and 10 Line 418 to 421<br>Emerald_Protocol_V2.0 28Jun2022 Page 3
	5b	Name and contact information for the trial sponsor<br>Main manuscript Page 10 Line 433<br>Emerald_Protocol_V2.0 28Jun2022 Page 3

For peer review only - http://bmjopen.bmj.com/site/about/guidelines.xhtml

	5c	Role of study sponsor and funders, if any, in study design; collection, management, analysis, and interpretation of data; writing of the report; and the decision to submit the report for publication, including whether they will have ultimate authority over any of these activities<br>None:<br>Emerald_Protocol_V2.0 28Jun2022 Page 38 Section 18.1
	5d	Composition, roles, and responsibilities of the coordinating centre, steering committee, endpoint adjudication committee, data management team, and other individuals or groups overseeing the trial, if applicable (see Item 21a for data monitoring committee)<br>Emerald_Protocol_V2.0 28Jun2022 Page 38 Section 18 and page 39 section 19
**Introduction**		
Background and rationale	6a	Description of research question and justification for undertaking the trial, including summary of relevant studies (published and unpublished) examining benefits and harms for each intervention<br>Main manuscript Page 3 Line 85-131<br>Emerald_Protocol_V2.0 28Jun2022 Page 14 and 15
	6b	Explanation for choice of comparators<br>Main manuscript page 3 116 to 131<br>Emerald_Protocol_V2.0 28Jun2022 Page 14 and 15
Objectives	7	Specific objectives or hypotheses<br>Main manuscript page 4 line 261-272<br>Emerald_Protocol_V2.0 28Jun2022 page 21
Trial design	8	Description of trial design including type of trial (eg, parallel group, crossover, factorial, single group), allocation ratio, and framework (eg, superiority, equivalence, noninferiority, exploratory)<br>Main manuscript page 4 line 169-170<br>Emerald_Protocol_V2.0 28Jun2022 page 19 and 20
**Methods: Participants, interventions, and outcomes**		
Study setting	9	Description of study settings (eg, community clinic, academic hospital) and list of countries where data will be collected. Reference to where list of study sites can be obtained<br>Main manuscript Page 6 line 250<br>Emerald_Protocol_V2.0 28Jun2022 page 22 and 23
Eligibility criteria	10	Inclusion and exclusion criteria for participants. If applicable, eligibility criteria for study centres and individuals who will perform the interventions (eg, surgeons, psychotherapists)<br>Main manuscript page 6 line 257 and Appendix 1<br>Emerald_Protocol_V2.0 28Jun2022 Page 21 and 22

For peer review only - http://bmjopen.bmj.com/site/about/guidelines.xhtml

Interventions	11a	Interventions for each group with sufficient detail to allow replication, including how and when they will be administered<br>Main manuscript page 6 line 276 to page 9 376 (radiotherapy planning, delineation, and treatment delivery)<br>Emerald_Protocol_V2.0 28Jun2022 page 29
	11b	Criteria for discontinuing or modifying allocated interventions for a given trial participant (eg, drug dose change in response to harms, participant request, or improving/worsening disease)<br>Main manuscript page 9 395-404<br>Emerald_Protocol_V2.0 28Jun2022 page 37
	11c	Strategies to improve adherence to intervention protocols, and any procedures for monitoring adherence (eg, drug tablet return, laboratory tests)<br>Main manuscript line Page 8 line 378-387<br>Emerald_Protocol_V2.0 28Jun2022 page 29
	11d	Relevant concomitant care and interventions that are permitted or prohibited during the trial<br>Main manuscript page 6 line 258-259<br>Emerald_Protocol_V2.0 28Jun2022 page 29
Outcomes	12	Primary, secondary, and other outcomes, including the specific measurement variable (eg, systolic blood pressure), analysis metric (eg, change from baseline, final value, time to event), method of aggregation (eg, median, proportion), and time point for each outcome. Explanation of the clinical relevance of chosen efficacy and harm outcomes is strongly recommended<br>Main manuscript page 6 line 263 to 273<br>Emerald_Protocol_V2.0 28Jun2022 page 21
Participant timeline	13	Time schedule of enrolment, interventions (including any run-ins and washouts), assessments, and visits for participants. A schematic diagram is highly recommended (see Figure)<br>Main manuscript page 4 line 196 to 198<br>Emerald_Protocol_V2.0 28Jun2022 page 9 and 10 and page 20
Sample size	14	Estimated number of participants needed to achieve study objectives and how it was determined, including clinical and statistical assumptions supporting any sample size calculations<br>Main manuscript page 9 line 391<br>Emerald_Protocol_V2.0 28Jun2022 page 35
Recruitment	15	Main manuscript page 3 Line 155-157<br>Emerald_Protocol_V2.0 28Jun2022 page 21-22

Methods: Assignment of interventions (for controlled trials)

Allocation:

Sequence generation	16a	Method of generating the allocation sequence (eg, computer-generated random numbers), and list of any factors for stratification. To reduce predictability of a random sequence, details of any planned restriction (eg, blocking) should be provided in a separate document that is unavailable to those who enrol participants or assign interventions<br>Main manuscript page 4 Line 173-194<br>Emerald_Protocol_V2.0 28Jun2022 page 20
Allocation concealment mechanism	16b	Mechanism of implementing the allocation sequence (eg, central telephone; sequentially numbered, opaque, sealed envelopes), describing any steps to conceal the sequence until interventions are assigned<br>n/a
Implementation	16c	Who will generate the allocation sequence, who will enrol participants, and who will assign participants to interventions<br>n/a
Blinding (masking)	17a	Who will be blinded after assignment to interventions (eg, trial participants, care providers, outcome assessors, data analysts), and how<br>n/a
	17b	If blinded, circumstances under which unblinding is permissible, and procedure for revealing a participant's allocated intervention during the trial<br>n/a

Methods: Data collection, management, and analysis

Data collection methods	18a	Plans for assessment and collection of outcome, baseline, and other trial data, including any related processes to promote data quality (eg, duplicate measurements, training of assessors) and a description of study instruments (eg, questionnaires, laboratory tests) along with their reliability and validity, if known. Reference to where data collection forms can be found, if not in the protocol<br>Main manuscript figure 2 Page 4 Line 196<br>Emerald_Protocol_V2.0 28Jun2022 page 33 and 34 and 38
	18b	Plans to promote participant retention and complete follow-up, including list of any outcome data to be collected for participants who discontinue or deviate from intervention protocols<br>Main manuscript Figure 2 Page 4 line 196<br>Emerald_Protocol_V2.0 28Jun2022 page 33,34,38 and 42

For peer review only - http://bmjopen.bmj.com/site/about/guidelines.xhtml

Data management	19	Plans for data entry, coding, security, and storage, including any related processes to promote data quality (eg, double data entry; range checks for data values). Reference to where details of data management procedures can be found, if not in the protocol<br>Emerald_Protocol_V2.0 28Jun2022 page 38 and 39
Statistical methods	20a	Statistical methods for analysing primary and secondary outcomes. Reference to where other details of the statistical analysis plan can be found, if not in the protocol<br>Main manuscript page 9 line 390 to 410<br>Emerald_Protocol_V2.0 28Jun2022 page 35-38
	20b	Methods for any additional analyses (eg, subgroup and adjusted analyses)<br>Emerald_Protocol_V2.0 28Jun2022 page 37
	20c	Definition of analysis population relating to protocol non-adherence (eg, as randomised analysis), and any statistical methods to handle missing data (eg, multiple imputation)<br>Emerald_Protocol_V2.0 28Jun2022 page 38
**Methods: Monitoring**		
Data monitoring	21a	Composition of data monitoring committee (DMC); summary of its role and reporting structure; statement of whether it is independent from the sponsor and competing interests; and reference to where further details about its charter can be found, if not in the protocol. Alternatively, an explanation of why a DMC is not needed<br>Trial Management Group (TMG) fulfil role of DMC<br>Emerald_Protocol_V2.0 28Jun2022 page 38
	21b	Description of any interim analyses and stopping guidelines, including who will have access to these interim results and make the final decision to terminate the trial<br>Main manuscript Page 9 Line 405 410<br>Emerald_Protocol_V2.0 28Jun2022 page 37
Harms	22	Plans for collecting, assessing, reporting, and managing solicited and spontaneously reported adverse events and other unintended effects of trial interventions or trial conduct<br>Main manuscript page 4 196 Figure 2<br>Emerald_Protocol_V2.0 28Jun2022 page 33 and 34
Auditing	23	Frequency and procedures for auditing trial conduct, if any, and whether the process will be independent from investigators and the sponsor<br>Emerald_Protocol_V2.0 28Jun2022 page 45
**Ethics and dissemination**		

Research ethics approval	24	Plans for seeking research ethics committee/institutional review board (REC/IRB) approval<br>Main manuscript page 2 line 56 to 58<br>Emerald_Protocol_V2.0 28Jun2022 Page 42 and 43
Protocol amendments	25	Plans for communicating important protocol modifications (eg, changes to eligibility criteria, outcomes, analyses) to relevant parties (eg, investigators, REC/IRBs, trial participants, trial registries, journals, regulators)<br>Emerald_Protocol_V2.0 28Jun2022 page 43
Consent or assent	26a	Who will obtain informed consent or assent from potential trial participants or authorised surrogates, and how (see Item 32)<br>Uploaded to scholar one as supplementary file<br>Emerald_Protocol_V2.0 28Jun2022 page 12
	26b	Additional consent provisions for collection and use of participant data and biological specimens in ancillary studies, if applicable<br>Emerald_Protocol_V2.0 28Jun2022 page 23
Confidentiality	27	How personal information about potential and enrolled participants will be collected, shared, and maintained in order to protect confidentiality before, during, and after the trial<br>Emerald_Protocol_V2.0 28Jun2022 page 45
Declaration of interests	28	Financial and other competing interests for principal investigators for the overall trial and each study site<br>Main manuscript page 10 line 438<br>Emerald_Protocol_V2.0 28Jun2022 page 1
Access to data	29	Statement of who will have access to the final trial dataset, and disclosure of contractual agreements that limit such access for investigators<br>Emerald_Protocol_V2.0 28Jun2022 page 38-41, 44 and 46
Ancillary and post-trial care	30	Provisions, if any, for ancillary and post-trial care, and for compensation to those who suffer harm from trial participation<br>Emerald_Protocol_V2.0 28Jun2022 page 20
Dissemination policy	31a	Plans for investigators and sponsor to communicate trial results to participants, healthcare professionals, the public, and other relevant groups (eg, via publication, reporting in results databases, or other data sharing arrangements), including any publication restrictions<br>Main manuscript page 2 line 56-59<br>Emerald_Protocol_V2.0 28Jun2022 page 46
	31b	Authorship eligibility guidelines and any intended use of professional writers<br>No intended us of professional writers<br>Main manuscript page 10 Lines 420-422

For peer review only - http://bmjopen.bmj.com/site/about/guidelines.xhtml

	31c	Plans, if any, for granting public access to the full protocol, participant-level dataset, and statistical code<br>Emerald_Protocol_V2.0 28Jun2022 will be available through ISRCTN registry public domain and through submitted for publication via BMJ_open
**Appendices**		
Informed consent materials	32	Model consent form and other related documentation given to participants and authorised surrogates<br>Uploaded as 'supplementary material' to scholar one Emerald_Protocol_V2.0 28Jun2022
Biological specimens	33	Plans for collection, laboratory evaluation, and storage of biological specimens for genetic or molecular analysis in the current trial and for future use in ancillary studies, if applicable<br>Emerald_Protocol_V2.0 28Jun2022 page 27

*It is strongly recommended that this checklist be read in conjunction with the SPIRIT 2013 Explanation & Elaboration for important clarification on the items. Amendments to the protocol should be tracked and dated. The SPIRIT checklist is copyrighted by the SPIRIT Group under the Creative Commons "Attribution-NonCommercial-NoDerivs 3.0 Unported" license.

For peer review only
